# Natural variations of *TFIIAγ* gene and *LOB1* promoter contribute to citrus canker disease resistance in *Atalantia buxifolia*

**DOI:** 10.1371/journal.pgen.1009316

**Published:** 2021-01-25

**Authors:** Xiaomei Tang, Xia Wang, Yue Huang, Ling Ma, Xiaolin Jiang, Muhammad Junaid Rao, Yuantao Xu, Ping Yin, Meng Yuan, Xiuxin Deng, Qiang Xu

**Affiliations:** 1 Key Laboratory of Horticultural Plant Biology Ministry of Education, Huazhong Agricultural University, Wuhan, the People's Republic of China; 2 Key Laboratory of Crop Genetic Improvement, National Center of Plant Gene Research, Huazhong Agricultural University, Wuhan, the People's Republic of China; University of Florida Institute of Food and Agricultural Sciences, UNITED STATES

## Abstract

Citrus canker caused by *Xanthomonas citri* subsp. *citri* (*Xcc*) is one of the most devastating diseases in citrus industry worldwide. Most citrus cultivars such as sweet orange are susceptible to canker disease. Here, we utilized wild citrus to identify canker-resistant germplasms, and found that *Atalantia buxifolia*, a primitive (distant-wild) citrus, exhibited remarkable resistance to canker disease. Although the susceptibility gene *LATERAL ORGAN BOUNDARIES 1* (*LOB1*) could also be induced in Atalantia after canker infection, the induction extent was far lower than that in sweet orange. In addition, three of amino acids encoded by transcription factor TFIIAγ in Atalantia (AbTFIIAγ) exhibited difference from those in sweet orange (CsTFIIAγ) which could stabilize the interaction between effector PthA4 and effector binding element (EBE) of *LOB1* promoter. The mutation of *AbTFIIAγ* did not change its interaction with transcription factor binding motifs (TFBs). However, the *AbTFIIAγ* could hardly support the *LOB1* expression induced by the PthA4. In addition, the activity of *AbLOB1* promoter was significantly lower than that of *CsLOB1* under the induction by PthA4. Our results demonstrate that natural variations of *AbTFIIAγ* and effector binding element (EBE) in the *AbLOB1* promoter are crucial for the canker disease resistance of Atalantia. The natural mutations of *AbTFIIAγ* gene and *AbLOB1* promoter in Atalantia provide candidate targets for improving the resistance to citrus canker disease.

## Author summary

It has been well documented that most citrus cultivars are susceptible to canker disease, while little is known about the resistance or susceptibility of primitive or wild citrus to canker disease. This study reveals that primitive citrus (*Atalantia buxifolia*) is highly resistant to citrus canker. Transcriptome data demonstrated that Atalantia had an active resistance response to the infection of *Xcc*, compared with susceptible sweet orange. Our results indicated that natural variations of *AbTFIIAγ* gene and *AbLOB1* promoter contributed to the resistance. Hence, we propose that the natural mutations of *AbTFIIAγ* gene and *AbLOB1* promoter could provide candidate targets for breeding canker resistant citrus.

## Introduction

Citrus canker is one of the most devastating bacterial diseases in citrus industry worldwide. It causes severe necrosis symptoms on the leaves and fruits, accelerating the dropping of citrus fruit and leaves [1]. This disease causes considerable yield losses, increases in bactericide cost, ecosystem risks, and even negative impacts on socio-economy [2]. One of economic and environment-friendly approaches is to improve the resistance of the host plants. The pathogenic bacteria of *Xanthomonas citri* strains harbor different host range. For example, XccA^W^ and XccA* strains have a narrow host range including Mexican lime, whereas XccA can cause disease in most commercial citrus varieties [3–5].

Since almost all citrus cultivars are susceptible to canker disease [6], there have been extensive studies of its pathogenesis. Previous studies have shown that *Xanthomonas* bacteria contain the type III secretion system (T3SS), and that different types of *Xanthomonas* strains have different numbers of type III secretion (T3S) effectors. Transcription activator-like effectors (TALEs) belong to the AvrBs3/PthA family of T3SS effectors, and they are vital for canker bacteria to form pustule on citrus [5,7]. A typical TALE processes an N-terminal translocation signal, a central repeat domain consisting of 34 amino acids, 3 nuclear localization signals, transcription factor binding motifs (TFBs), and C-terminal amino acid activation domain [8,9]. PthA4 of *Xcc* can activate LATERAL ORGAN BOUNDARIES 1 (*LOB1*) gene expression [10], and the expression of susceptibility genes can be suppressed either by variation of the TFIIAγ or by mutation of the effector binding element (EBE) [11]. *LOB1* is a transcription factor belonging to the *LATERAL ORGAN BOUNDARIES (LOB)* gene family, which not only controls the expansion and growth of plant cells, but also promotes bacterial growth and pustule formation in citrus [12,13].

Transcription factor IIA (TFIIA) is vital for the transcriptional regulation in eukaryotes. In humans, TFIIA contains three subunits, including α, β and γ subunits, among which α and β subunits are encoded by the same gene, while in yeast, TFIIA only contains the TOA1 and TOA2 subunits with TOA2 homologous to human *TFIIAγ* subunit [14,15]. Several studies have illustrated that TFIIA is required for RNA polymerase II-dependent transcription, and it can stabilize the TATA box-binding protein (TBP)-TFIID complex in the TATA box region of the promoters [16–18]. In plants, *TFIIAγ* is expressed in most tissues, especially in the young and active tissues [19]. In addition, *TFIIAγ* is associated with the disease resistance of rice [20]. In rice, it has been reported that TFBs are found to be vital for the infection of *Xanthomonas* bacteria, that TFBs can interact with the host basal transcription factor IIA gamma subunit (OsTFIIAγ5) to promote the expression of susceptibility genes, and that silencing *OsTFIIAγ5* results in the increased resistance to *Xanthomonas oryzae* pv. *oryzae* (*Xoo*) and *Xanthomonas oryzae* pv. *oryzicola* (*Xoc*) [20]. However, its allele xa5, which carries a V to E mutation at the 39^th^ amino acid position, could not interact with the TFBs of *Xoo* and *Xoc* [21,22]. The mutation at the 39^th^ amino acid could lead to the interaction of xa5 with an acidic activation domain (AAD) of avirulence protein Avrxa5, thus impeding host cell transcription, eventually resulting in subsequent cell death as well as the resistance reaction [23,24]. In sweet orange, TALE TFBs could also interact with basal transcription factor (CsTFIIAγ) to promote *Xcc* infection [20].

In rice, *SWEET* genes belong to the sucrose transporter family, and are required for the susceptibility to rice blight. Natural mutations in the EBEs of *SWEET* genes have been reported in rice blight resistant varieties [11,25]. Editing the EBE regions of TALE-induced *SWEET* genes impedes the expression of corresponding susceptibility genes [26]. To the best of our knowledge, the polymorphism of the EBEs in the susceptibility gene promoter has not been reported in citrus canker resistant varieties so far.

Citrus has a broad spectrum of resources ranging from primitive, wild, and cultivated varieties. Currently, little is known about the resistance or susceptibility of primitive or wild citrus to canker disease. *Atalantia buxifolia* (Chinese box orange), as a primitive citrus, shows tolerance to diverse abiotic and biotic stresses and is sometimes used as rootstock for the grafting of commercial citrus [27,28]. It becomes possible to identify resistant genes to citrus canker disease with the availability of the genomes of Atalantia and other wild germplasms [29,30]. In this study, we found that Atalantia is resistant to citrus canker. The susceptibility gene *LOB1* was induced in Atalantia after inoculation with *Xcc*, but its induction degree was much lower in Atalantia than in sweet orange. In contrast to *CsTFIIAγ* of sweet orange, *AbTFIIAγ* of Atalantia could hardly support the expression of the *CsLOB1* gene induced by PthA4, and the activity of the *AbLOB1* promoter was significantly lower than that of *CsLOB1* induced by PthA4. Our results indicate that the natural mutations of *AbTFIIAγ* gene and *AbLOB1* promoter are vital for the canker resistance of Atalantia.

## Results

### Resistance of Atalantia to citrus canker

**Fig 1:**
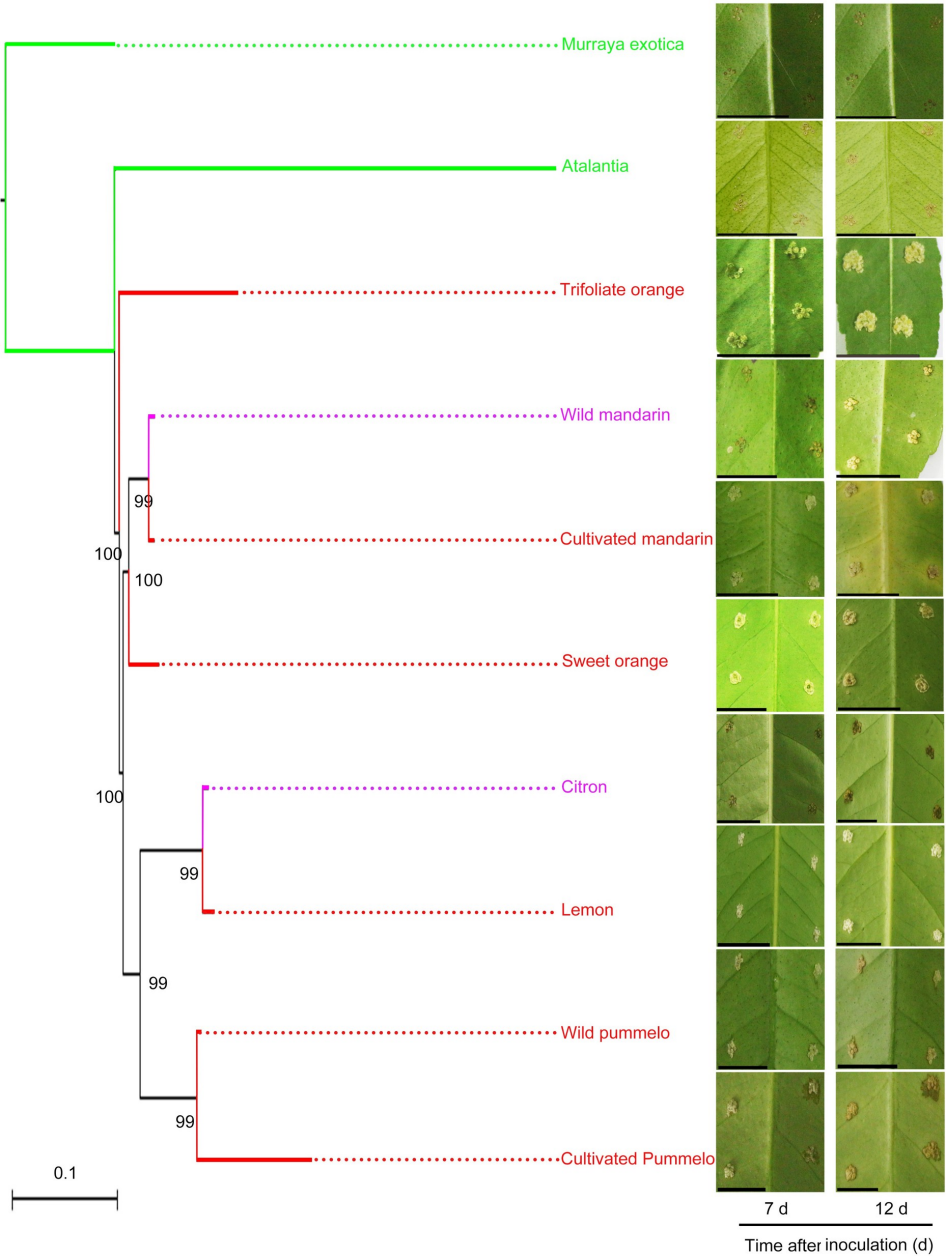
*Atalantia buxifolia* resistance to citrus canker. The phylogenetic tree was constructed by using the maximum likelihood tree method and the substitution model GTRGAMMA in the RAxML software. *Murraya koenigii* was used as the outgroup. Fully expanded leaves were treated with *Xcc* (10^8^ CFU/mL) to evaluate citrus canker resistance. The symptoms were observed at 7 and 12 d after inoculation. Red indicates canker susceptible citrus (Wild pummelo: purple pummelo; Cultivated pummelo: guanxi pummelo; Cultivated mandarin: Ponkan; Lemon: Eureka lemon). Purple indicates canker tolerant citrus (Wild mandarin: mangshan mandarin). Green indicates canker-resistant citrus. Bootstrap values greater than 60 are labelled at the node in the tree. Scale bars, 1 cm.

Great research efforts have been made to evaluate the canker disease resistance of various commercial citrus cultivars and the relatives, and almost all cultivars have been found to be susceptible to the disease [2,7]. To explore the canker resistant germplasms, we expanded the research scope to investigate wild citrus in tribe Citrinae belonging to the subfamily Aurantioideae. Ten citrus varieties were chosen for canker disease evaluation. The disease lesion area was determined at 12 d after the inoculation with *Xcc*. Based on the results, the citrus varieties were classified into three categories: susceptible (lesion area = 2–4 mm^2^), tolerant (lesion area = 1–2 mm^2^), and resistant (lesion area = 0–1 mm^2^) (S1 Fig). Three wild citrus varieties including wild mandarin, wild pummelo, and citron showed different degrees of susceptibility to citrus canker. Wild mandarin and citron showed tolerance to citrus canker in the early stages, but exhibited small lesions at 12 d after inoculation. The wild pummelo of purple pummelo were susceptible to citrus canker. However, remarkable resistance was observed in *Atalantia buxifolia*, a distant wild relative of citrus (Fig 1).

**Fig 2:**
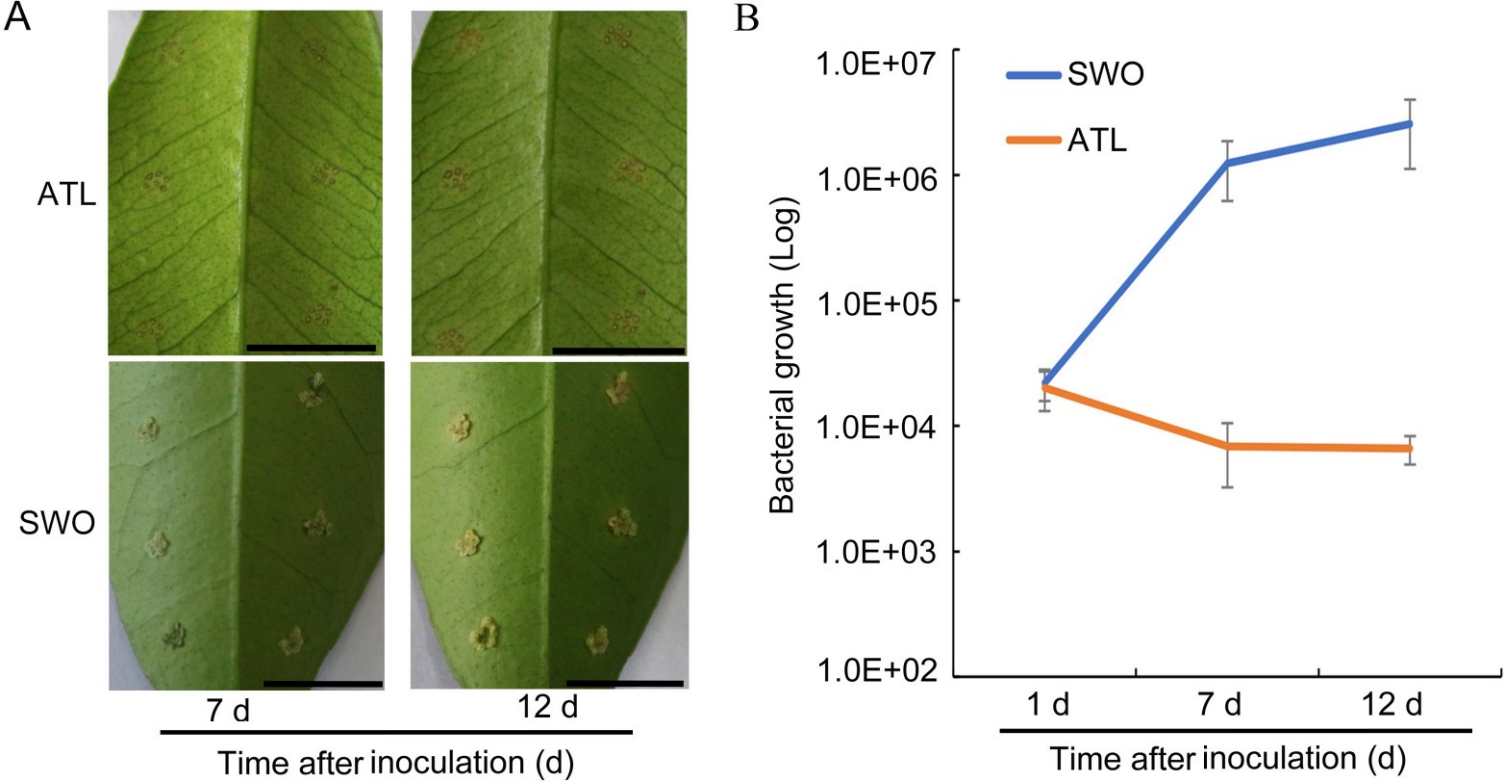
Inability to grow of *Xcc* in Atalantia at low bacterial titer. (A) Fully expanded mature leaves of Atalantia (ATL) and sweet orange (SWO) were treated with *Xcc* (10^6^ CFU/mL). Photographs were taken at 7 and 12 d after inoculation. (B) Bacterial growth in ATL and SWO at 1, 7, and 12 d after *Xcc* inoculation. Error bars indicate standard deviation of three independent tests. Scale bars, 1 cm.

A low bacterial titer of *Xcc* was used to further evaluate the canker disease development in Atalantia. Sweet orange, a variety known to be susceptible to canker disease, was used as the control. Canker development was assessed at 7 d and 12 d after *Xcc* inoculation (Fig 2). At 7 d, canker lesion was not observed on the leaves of Atalantia, but obvious canker symptoms appeared on the leaves of sweet orange (Fig 2A). Bacterial growth evaluation results showed that the population of *Xcc* decreased at 7 and 12 d in Atalantia, compared with that in sweet orange, indicating the inhibition of bacterial growth (Fig 2B).

### Differentially expressed genes of Atalantia in response to canker infection

**Fig 3:**
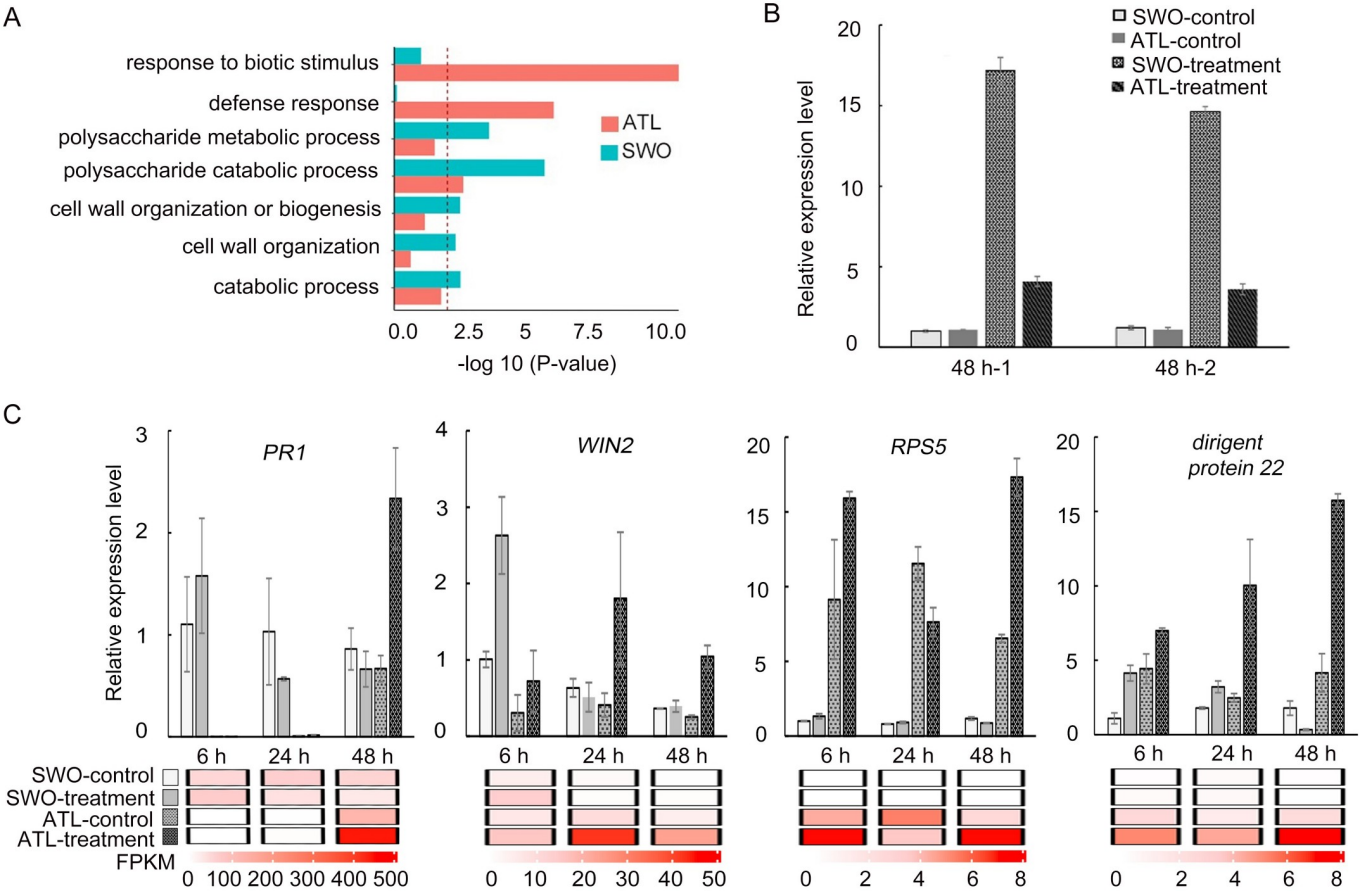
Differentially expressed genes in Atalantia and sweet orange after *Xcc* (10^8^ CFU/ml) inoculation. (A) GO enrichment analysis of the up-regulated genes in Atalantia (ATL) and sweet orange (SWO) at 48 h after inoculation. (B) qRT-PCR validation of *LOB1* expression in Atalantia and sweet orange at 48 h after inoculation with *Xcc* and sterile water. (C) Expression levels of four DEGs: pathogenesis-related gene 1 (*PR1*), Resistance to *Pseudomonas syringae* 5 (*RPS5*), HopW1-1-Interacting 2 (*WIN2*), and dirigent-like protein 22 genes, and their corresponding expression patterns in RNA-Seq data in Atalantia and sweet orange. The treatment group was inoculated with *Xcc* and the control group was inoculated with sterile water at 6 h, 24 h, and 48 h. All the gene expressions were normalized according to the gene expression of the sweet orange control group at 6 h post inoculation. The relative expression level was calculated by 2^-△△Ct^ method with *EF1a* as the reference gene. Error bars indicate standard deviation of three independent repetitions.

To reveal the molecular basis for the resistance of Atalantia to *Xcc*, samples were collected from leaves inoculated with *Xcc* (treatment) and leaves inoculated with sterile water (control) at different time points (hour 6, 24 and 48). A total of approximately 772 million paired-end reads (32 million reads for each library on average) were obtained by Illumina sequencing technology (S1 Table). Gene ontology (GO) analysis results indicated that the up-regulated genes in Atalantia were enriched in pathways such as defense response and biotic stimulus response, while those in sweet orange were enriched in polysaccharide metabolic and catabolic pathways at 48 h after the inoculation (Fig 3A). The cross-comparison Venn diagram of differentially expressed genes (DEGs) showed that eight DEGs were overlapped between Atalantia and sweet orange, one of which was citrus canker susceptibility gene *LOB1* (S2 Table) [10]. However, the transcriptome data showed that the relative expression level of *AbLOB1* was much lower than that of *CsLOB1*. Quantitative real-time PCR data confirmed that the expression level of *CsLOB1* was three folds as high as that of *AbLOB1* (Fig 3B). To confirm expression of the DEGs (S3 Table), quantitative RT-PCR was conducted for 10 candidate genes chosen from the transcriptional profile. These unigenes such as pathogenesis-related 1 (*PR1*), HopW1-1-interacting 2 (*WIN2*), dirigent-like protein 22, and pathogenesis-related 4 (*PR4*) were up-regulated in Atalantia from 6 h to 48 h after *Xcc* inoculation, while these genes were down-regulated in sweet orange. Gene *RPS5* (Resistant to *pseudomonas syringae* 5) and quinone oxidoreductase gene were only significantly induced in Atalantia at 6 h, 24 h, 48 h after *Xcc* inoculation. Other genes such as repressor GA3 (*RGA3*), cytochrome P450 (*CYP76C4*) were up-regulated in Atalantia and down-regulated in sweet orange at 48 h after *Xcc* inoculation (Figs 3C and S2). Correlations between the ten genes expression and RNA-seq data were shown in S2C Fig. Overall, the expression profile of most genes selected for qRT-PCR validation was consistent with the RNA-Seq data (S2C Fig).

### Genetic variations of *TFIIAγ* in resistant and susceptible citrus and their interaction with TAL effectors

**Fig 4:**
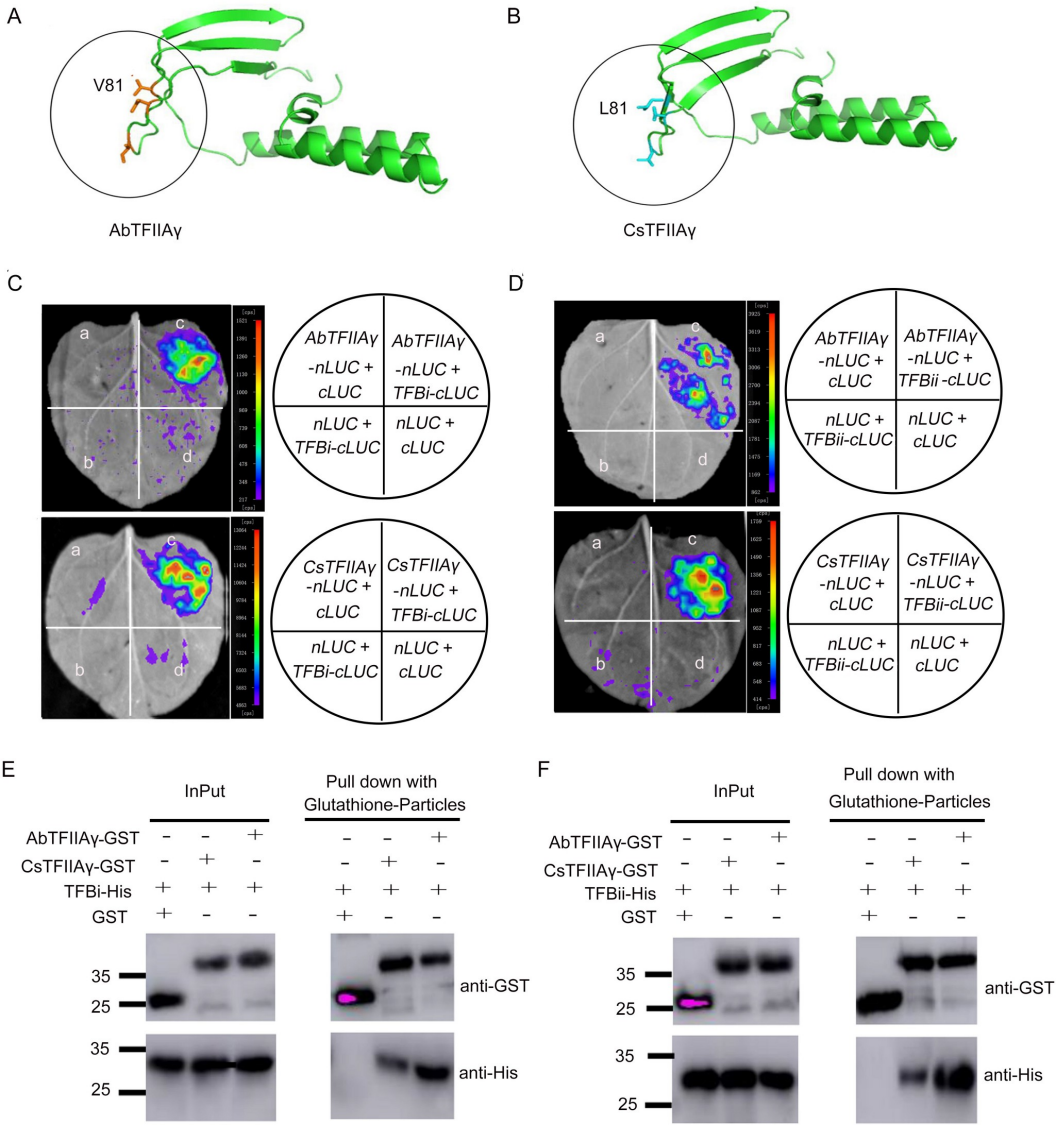
Interactions of AbTFIIAγ or CsTFIIAγ with TALE TFBs. (A) Homology modeling of AbTFIIAγ protein. (B) Homology modeling of CsTFIIAγ protein. The structures of TFIIAγ proteins were homology modeled using SWISS-MODEL online service with default parameters (Fig 4A and 4B). (C) Interactions between AbTFIIAγ-nLuc or CsTFIIAγ-nLuc and TFBi-cLuc. (D) Interactions between AbTFIIAγ-nLuc or CsTFIIAγ-nLuc and TFBii-cLuc. Note: a, b, and d indicate the negative controls of tobacco leaves; c denotes the interactions between TFBi-cLuc or TFBii-cLuc and AbTFIIAγ-nLuc or CsTFIIAγ-nLuc in tobacco leaves in luciferase complementation assay (Fig 4C and 4D). (E) The interaction of AbTFIIAγ or CsTFIIAγ with TFBi in pull-down assays. (F) The interaction of AbTFIIAγ or CsTFIIAγ with TFBii in Pull-down assays. Note: GST-tagged AbTFIIAγ or GST-tagged CsTFIIAγ and His-tagged TFBi or His-tagged TFBii were incubated with immobilized glutathione S-transferase (GST) (Fig 4E and 4F). The experiments were repeated three times independently.

Previous studies have shown that *TFIIAγ* could stabilize TAL effectors in the TATA box region and induce *LOB1* expression and susceptibility [20]. We further investigated the sequence variations of the *TFIIAγ* gene in the resistant and susceptible germplasms. Full-length nucleotide sequences of *TFIIAγ* were cloned from primitive, wild, and cultivated varieties. Amino acid alignment indicated that the TFIIAγ was highly conserved in the investigated varieties except the resistant citrus Murraya and Atalantia from primitive varieties, and that Murraya and Atalantia showed slight differences in amino acid sequences from susceptible and tolerant citrus (S3 Fig). Furthermore, the three-dimensional structures of AbTFIIAγ and CsTFIIAγ protein were modeled. Homology modeling of AbTFIIAγ and CsTFIIAγ proteins (Fig 4A and 4B) and their mutant proteins (S4 Fig) revealed that the 81^st^ residue of AbTFIIAγ and CsTFIIAγ was exposed on the surface of a β-sheet, and that the side chain of leucine (L81) in CsTFIIAγ was longer than that of valine (V81) in AbTFIIAγ.

To determine whether AbTFIIAγ can interact with TALE TFBs, luciferase complementation and pull-down assay were carried out. Luciferase activity was detected when AbTFIIAγ-nLuc or CsTFIIAγ-nLuc was co-expressed with TFBi-cLuc/TFBii-cLuc (Fig 4C and 4D), indicating both AbTFIIAγ and CsTFIIAγ could interact with TFBs. GST pull-down assay in vitro was performed using purified AbTFIIAγ-GST/CsTFIIAγ-GST and TFBi-His/TFBii-His proteins to further verify their interaction. Pull-down assay results indicated that His-tagged TFBi or TFBii interacted with the GST-tagged AbTFIIAγ and CsTFIIAγ proteins, but not with GST alone (Fig 4E and 4F).

### Enhancement of resistance to *Xcc* by silencing of *TFIIAγ* in sweet orange

**Fig 5:**
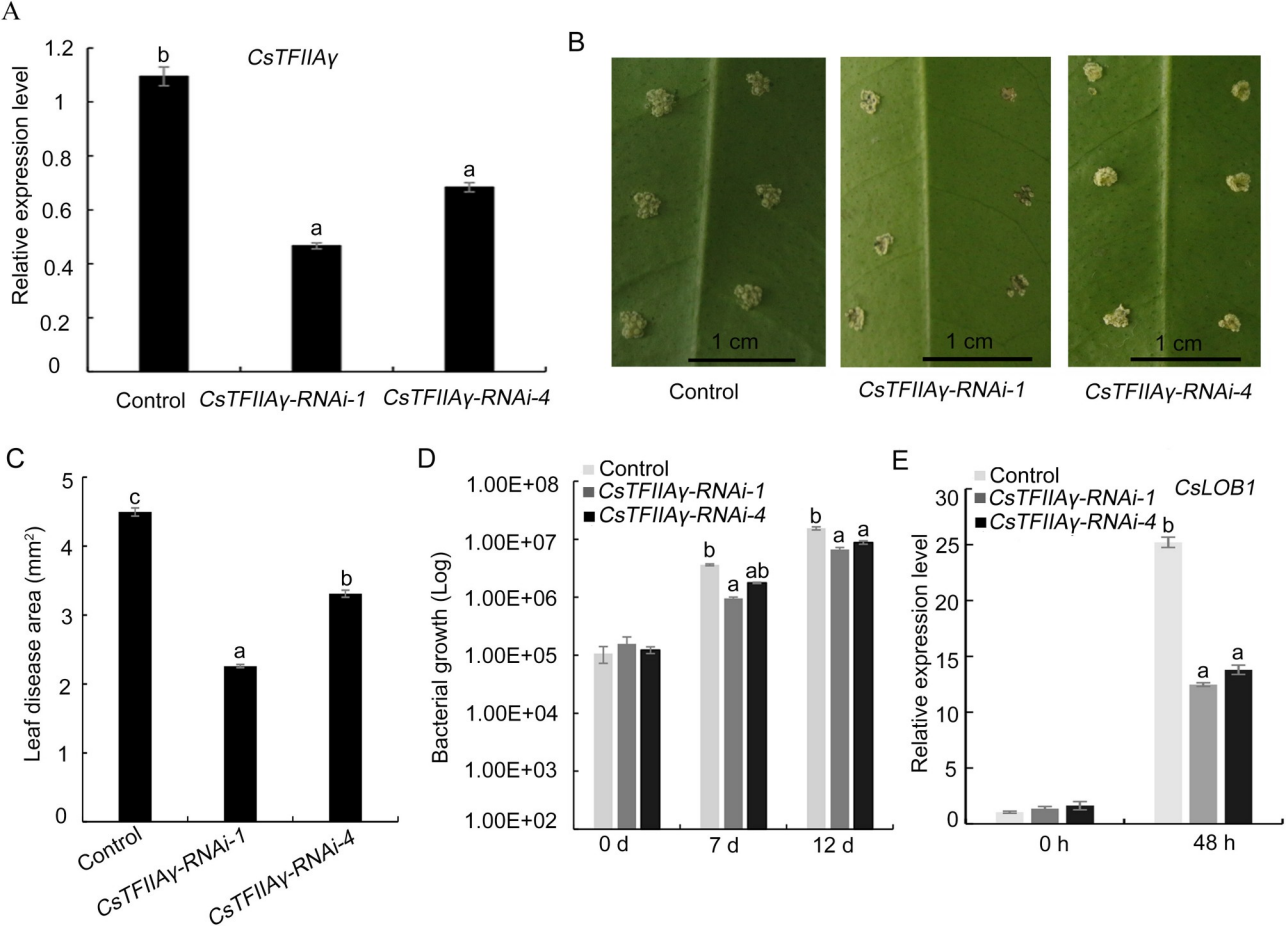
Enhancement of sweet orange resistance to *Xcc* through *CsTFIIAγ* RNA interference. (A) Relative expression levels of *CsTFIIAγ* in *CsTFIIAγ-RNAi* group and control group. (B) Symptoms of *CsTFIIAγ-*silenced lines (RNAi-1 and RNAi-4) and control leaves after inoculation with *Xcc* (10^8^ CFU/mL). The photos were taken at 12 d after inoculation. (C) Disease lesion area on *CsTFIIAγ*-silenced lines and control leaves at 12 d after inoculation. The disease lesion area was calculated by ImageJ 2.0. (D) Bacterial growth in *CsTFIIAγ*-RNAi plants and control plants at 0, 7, and 12 d after inoculation. (E) Relative expression level of *CsLOB1* at 48 h after inoculation. Data from three independent replicates were expressed as mean ± SD. Different letters above the bars represent significant differences (P < 0.05) in Duncan’s multiple range test.

A previous report has revealed that transient interference of *CsTFIIAγ* in sweet orange enhanced its resistance to *Xcc* [20]. Here, we suppressed the *CsTFIIAγ* gene expression in sweet orange leaves by RNA interference (RNAi) for the functional complementation of *AbTFIIAγ* because of the unavailability of the gene transformation system for Atalantia. The results showed that the reduction of *CsTFIIAγ* gene expression resulted in the decrease in the disease lesion area and the inhibition of the bacterial growth and the *CsLOB1* expression, compared with the control (Fig 5).

### Complementation assay of *AbTFIIAγ* and *CsTFIIAγ*

**Fig 6:**
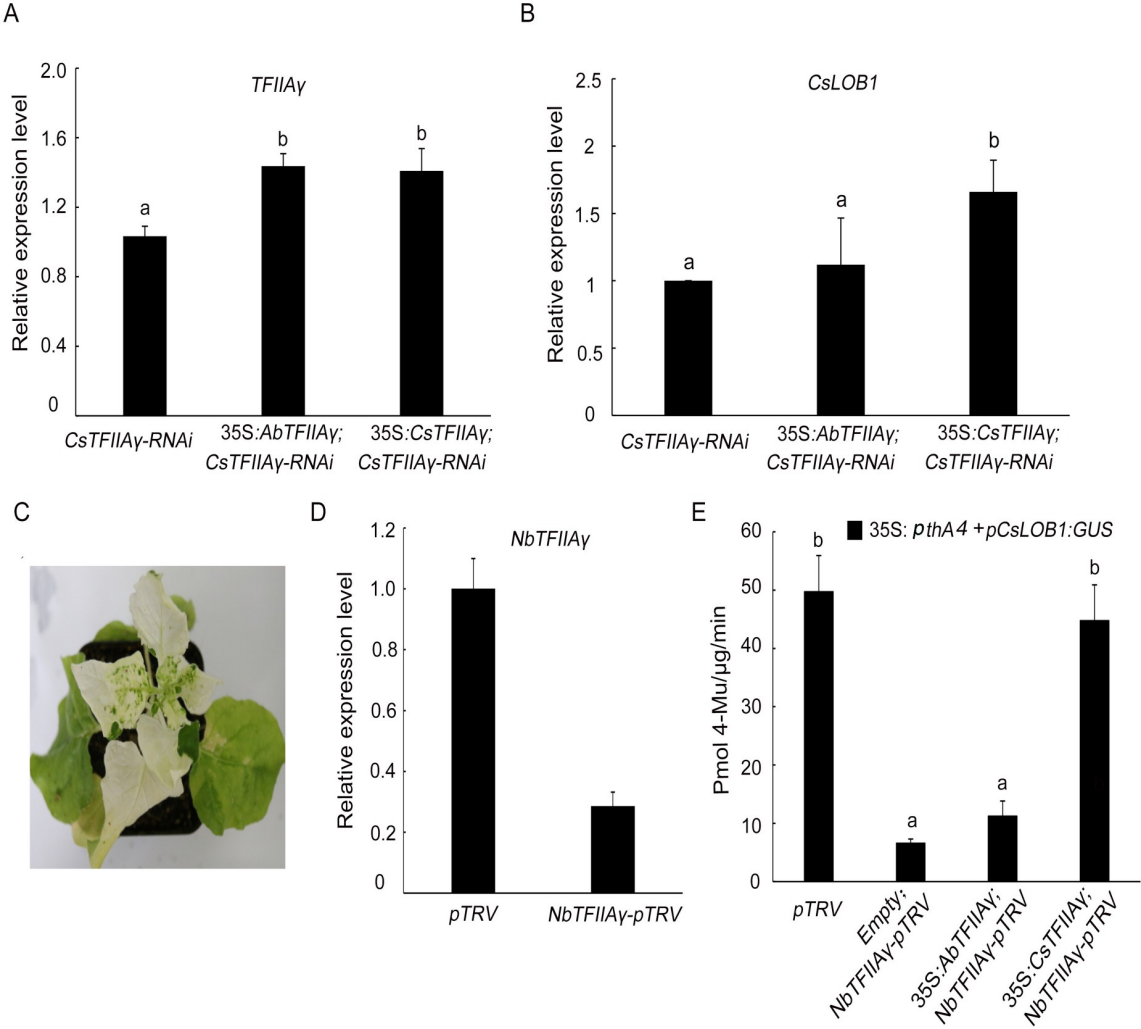
*CsLOB1* gene expression in *TFIIAγ-*silenced sweet orange and tobacco hardly supported by *AbTFIIAγ*. (A) Relative expression of *TFIIAγ*. (B) Relative expression of *CsLOB1*. Transient expression of *AbTFIIAγ*, *CsTFIIAγ*, and empty vector in *CsTFIIAγ-RNAi* sweet orange line (RNAi-1) at 4 days after *Xcc* (10^8^ CFU/ml) inoculation (Fig 6A and 6B). (C) Albino phenotype exhibited by *NbPDS-pTRV* tobacco at 15 d after silencing *NbPDS*. (D) Relative expression level of *NbTFIIAγ* in *NbTFIIAγ-pTRV* and control (*pTRV*) tobacco. (E) Expression of vector *pCsLOB1*:*GUS* induced by 35S:*pthA4* in *NbTFIIAγ-pTRV* plants after complementation of *AbTFIIAγ*, *CsTFIIAγ*, or empty vector. Data from three independent replicates were expressed as mean ± SD. Different letters above the bars represent significant differences (P < 0.05) in Duncan’s multiple range test.

*AbTFIIAγ* or *CsTFIIAγ* was transformed into a vector containing the promoter CaMV 35S and terminator NOS, respectively. Subsequently, the resultant two vectors were respectively transformed into *CsTFIIAγ-*silenced citrus line (RNAi-1). Complementary *CsTFIIAγ* in RNAi-1 line treated with *Xcc* promoted the *CsLOB1* gene expression, whereas the complementary *AbTFIIAγ* hardly supported this gene expression (Fig 6A and 6B). In order to further clarify the function of *AbTFIIAγ*, we performed the complementary assay in *Nicotiana benthamiana*. First, *N*. *benthamiana TFIIAγ* (*NbTFIIAγ*) was silenced, and the *N*. *benthamiana* phytoene dehydrogenase gene was used as the positive control to confirm the effectiveness of the virus-induced gene silencing (VIGS). Obvious albino phenotype was observed at 15 d (Fig 6C). *CsLOB1* promoter fused with the β-glucuronidase (GUS) reporter gene vector and 35S promoter-driven *pthA4* vector were transformed into *NbTFIIAγ-*silenced tobacco to detect the function of *AbTFIIAγ* and *CsTFIIAγ*. We first confirmed that gene *AbTFIIAγ* and *CsTFIIAγ* were expressed in *NbTFIIAγ-*silenced tobacco (S5 Fig), then we checked the promoter activity. The results showed that the complementary *CsTFIIAγ* restored the reduced GUS activity of *CsLOB1* promoter in *NbTFIIAγ-*silenced plants, while the complementary *AbTFIIAγ* hardly restored the reduced activity of *CsLOB1* promoter which was similar to that of complementary empty vector (Fig 6D and 6E).

### Significant lower promoter activity of *AbLOB1* than that of *CsLOB1* mediated by PthA4

**Fig 7:**
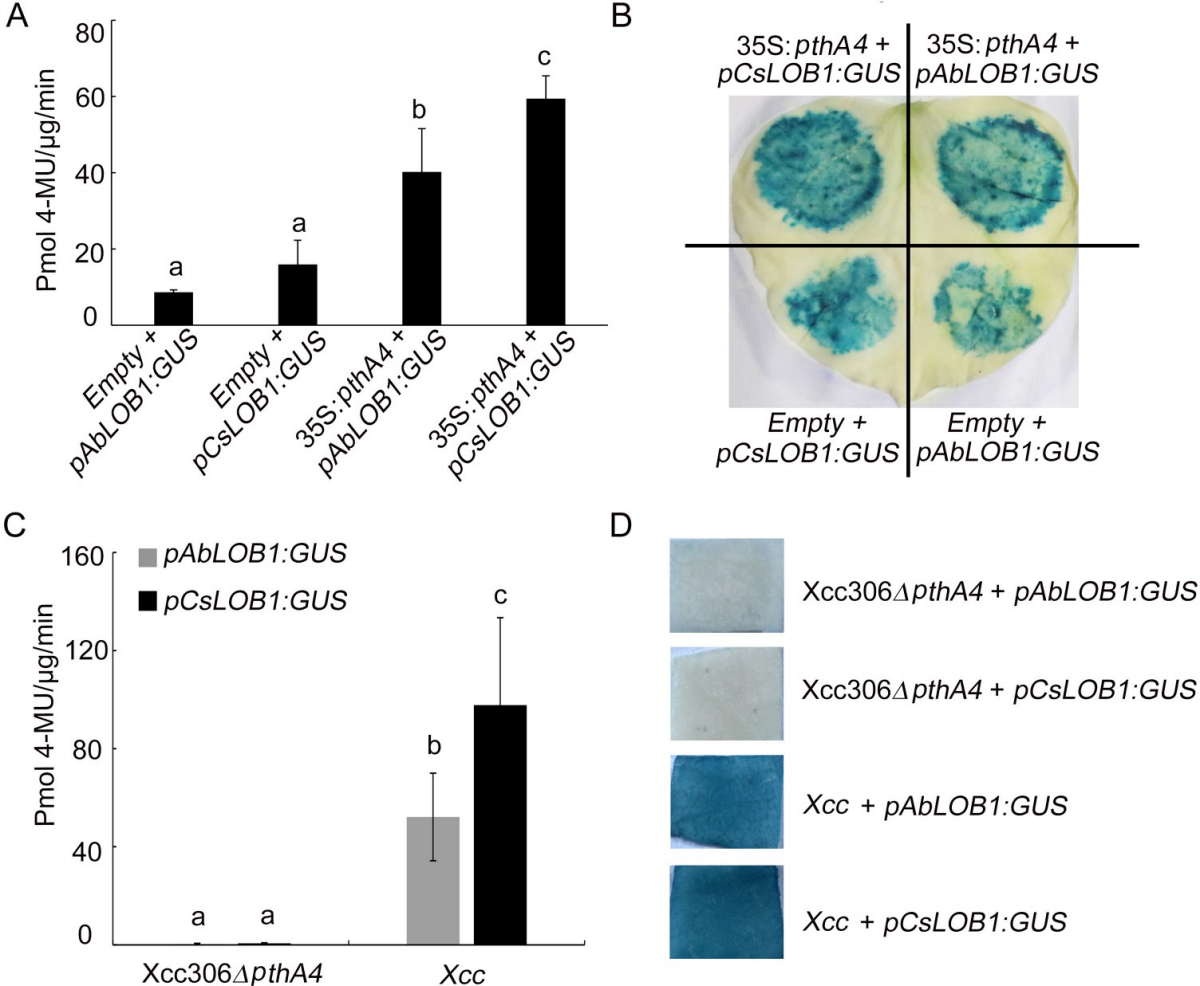
Significantly lower promoter activity of *AbLOB1* than *CsLOB1* induced by PthA4. (A) Promoter activity of *AbLOB1* and *CsLOB1* induced by PthA4 in tobacco. (B) GUS staining assay in tobacco leaves. *Agrobacteria* containing *pAbLOB1*:*GUS* or *pCsLOB1*:*GUS* mixed with 35S: *pthA4* or empty vector were infiltrated into tobacco leaves. Samples were collected at 2 days (Fig 7A and 7B). (C) Transient GUS activity related to *pAbLOB1* and *pCsLOB1* promoters after inoculation with *Xcc* or Xcc306*ΔpthA4* in sweet orange. Samples were collected for further analysis at 4 days post inoculation. (D) GUS staining assay of sweet orange leaves upon ectopic expression of *pAbLOB1*:*GUS* or *pCsLOB1*:*GUS* post inoculation with *Xcc* or Xcc306*ΔpthA4*. Error bars indicate standard deviation of three independent replicates. Different letters above the bars represent significant differences (P < 0.05) in Duncan’s multiple range test.

Previous research has shown that the PthA4 of *Xcc306* can bind to the promoter of *LOB1* to increase its expression [10]. We further compared *AbLOB1* and *CsLOB1* promoters and coding sequences, and found two single nucleotide polymorphisms (SNPs) in their EBE regions of promoters (S6A Fig). Amino acid alignment revealed that LOB1 was highly conserved (S6B Fig). To further verify the activities of the two promoters, *AbLOB1* and *CsLOB1* promoters were fused to pKGWFS7 vector containing the β-glucuronidase (GUS) reporter gene [10]. No significant difference in GUS activity was observed between *AbLOB1* or *CsLOB1* co-infiltrated with empty vector PBI121, but the GUS activity of *AbLOB1* was significantly lower than that of *CsLOB1* when these two promoters were co-infiltrated with vector 35S:*pthA4* (Fig 7A and 7B). We further compared the activities of these two promoters in sweet orange leaves by transient expression mediated by *Xcc* [10]. The results showed that GUS activity of *AbLOB1* was also significantly lower than that of *CsLOB1* when these two promoters were co-infiltrated with the *Xcc*, whereas no GUS activity of two promoters was observed upon the co-infiltration with the mutant strain Xcc306*ΔpthA4* (Fig 7C and 7D).

## Discussion

It has been reported that cultivated citrus varieties are more susceptible to canker disease than wild citrus, and that wild citrus varieties have a broad spectrum of disease resistance, whereas cultivated citrus varieties have lost certain resistance to different degrees during domestication [2,6]. For example, Meiwa kumquat obtain a durable resistance to *Xcc* by upregulating a host susceptibility gene to elicit immune responses [31]. Tracing back to wild germplams is an alternative strategy for identifying gene resources for breeding purpose. Chinese box orange (*Atalantia buxifolia*) is widely spread in Southern China, Philippines, and Japan [32,33]. It is a distant wild relative of *Citrus*, and it is occasionally used as rootstock due to its tolerance to diverse biotic and abiotic stresses [28]. In this study, we demonstrated that Atalantia, as a wild variety, was resistant to citrus canker.

*LOB1* is a susceptibility gene whose expression is crucial for the formation of pustule in different citrus varieties [10,12]. We found that the expression of *LOB1* was remarkably lower in Atalantia than in sweet orange. Previous studies have indicated that *TFIIAγ* can stabilize and increase the expression of the *LOB1* gene [20]. Considering this, we examined the genetic variations of *TFIIAγ* in resistant and susceptible varieties, and found that natural mutations in Atalantia, AbTFIIAγ could hardly support TALE-mediated *CsLOB1* gene expression (Fig 6). Homology modeling of AbTFIIAγ and CsTFIIAγ proteins and their mutant proteins revealed that the 81^st^ residues of AbTFIIAγ and CsTFIIAγ were exposed on the surface of a β-sheet, and that the side chain of leucine (L81) was longer than that of valine (V81). We speculated that leucine (L81) in sweet orange might be more conducive to the stabilization of the protein complex (TBP)-TFIID in the TATA box region of *LOB1* promoter due to its longer alkyl side chain than valine (V81) in Atalantia, which may partly explain the difference expression of *LOB1* in sweet orange and Atalantia. Based on the above results, it could be concluded that Atalantia possessed an unique allele *AbTFIIAγ*, which contributed to the canker resistance in Atalantia.

The mutation of EBE on the *AbLOB1* promoter also contributed to the disease resistance in Atalantia. We found two substitutions at the 7^th^ (C to T) and 13^th^ (T to C) position in the EBE region of promoter *AbLOB1* (S6A Fig). A previous study has shown that a single base insertion in the EBE region could offset PthA4-mediated *CsLOB1* expression in citrus, and that the substitution at the 9^th^ (C to T) position could compromise PthA4-induced *CsLOB1* expression [10]. Consistently, a substitution at the 10^th^ position in the EBE region appeared to compromise AvrXa7-induced *OsSWEET14* expression in a study of rice blight [25]. Our results revealed that the natural mutation of EBE on the *AbLOB1* promoter also compromised PthA4-induced *LOB1* expression (Fig 7). To our knowledge, the expression of *LOB1* is crucial for the formation of pustule. A comparison of *LOB1* gene expression 48 h after inoculation with *Xcc* or Xcc306*ΔpthA4* in Atalantia and sweet orange revealed that the expression of *AbLOB1* inoculated with *Xcc* was similar to that of *CsLOB1* inoculated with Xcc306*ΔpthA4* (S7 Fig). It is known that the Xcc306*ΔpthA4* strain is not pathogenic in citrus, and that low expression of *LOB1* can reduce the formation of pustule [10]. On the other hand, the inability of *Xcc* to grow on the leaf of Atalantia (Fig 2B) and the up-regulation of the disease defense-related genes (Figs 3C and S2) suggested that an active response occurred in Atalantia after *Xcc* infection.

Our data demonstrated that both cis- and trans-regulatory elements of *AbLOB1* contributed to the canker resistance of Atalantia. The mutations might provide some useful target sites for improving the resistance to citrus canker, a devastating and stubborn disease which is mostly controlled by chemicals at present [34,35]. The current control approaches are costly due to the use of bactericide, and environmentally- and ecologically-unfriendly [36,37]. Breeding canker-resistant cultivars is a promising strategy for solving the above problems. Recently, genome editing technology has been widely applied to breeding disease-resistant cultivars due to its high efficiency and accuracy. In rice, *SWEET* genes are the members of sucrose transporter gene family, and are required for the susceptibility to rice blight. Gene editing in the EBE regions of *SWEET11*, *SWEET13*, and *SWEET14* promoters could confer rice with broad-spectrum resistance to bacterial blight [38]. In citrus, editing the susceptibility gene *CsLOB1* and its promoter could enhance citrus canker resistance. However, most of the edited citrus might still be infected with canker disease to different degrees [39,40]. Our data indicated the *CsTFIIAγ* could regulate the expression of gene *CsLOB1*. It is worthwhile to evaluate the phenotype and agronomic performances of citrus plants whose *TFIIAγ* gene and EBEs of *LOB1* are edited. Homology recombination [41,42] and single base editing [43] are promising approaches to simultaneously modifying *TFIIAγ* and EBEs of *LOB1* in susceptible citrus.

## Experimental procedures

### Materials and methods

#### Plant materials and pathogen

All the citrus varieties used in this study were taken from the National Citrus Breeding Center of Huazhong Agricultural University. All the transgenetic and control plants were grown in a greenhouse at 25–30°C. The *X*. *citri* strain 3213 (hereafter referred to as *Xcc*) and the mutant strain Xcc306*ΔpthA4* were grown on nutrient agar medium at 28°C as previously reported [44].

#### Assay of pathogenicity

Fully expanded leaves were inoculated with *Xcc* (10^6^ and 10^8^ CFU/ml) and Xcc306*ΔpthA4* (10^8^ CFU/ml) with an inoculating needle (0.5 mm in diameter). Each inoculation site consisted of six pricks according to previous reports [45] with minor modifications. Bacterial suspension was dropped into each inoculation site. The disease lesion area was measured (36 punctures on average) with ImageJ 2.0. To examine the bacterial growth in citrus plants, leaf disk with 0.5 cm diameter in inoculated area was punched at 0, 1, 7, and 12 d after inoculation. DNA was extracted from 100 mg fresh weight of inoculated leaf disks, and 100 ng of total nucleic acid per inoculated sample was used as the template for quantitative polymerase chain reaction (qPCR). *pthA* of *Xcc* was used to calculate the bacterial population using the following formula [46,47].

$$\mathrm{Copy}\;\mathrm{Number}\;(Xcc,\;pthA)=10^{\left(38.3-ct\right)/3.56}.$$

#### Construction of phylogenetic tree of Citrinae

SNPs contained in the conserved single-copy genes in citrinae genomes were used for the construction of the phylogenetic tree of 10 varieties, namely, Murraya exotica, Atalantia, Citron, Wild mandarin (mangshan mandarin), Cultivated mandarin (Ponkan), Trifoliate orange, Lemon (Eureka lemon), Wild pummelo (purple pummelo), Cultivated pummelo (guanxi pummelo), Sweet orange. The maximum likelihood tree was constructed by using the substitution model GTRGAMMA in the RAxML software with *Murraya koenigii* as the outgroup. A total of 1,000 rapid bootstrap inferences were performed. The bootstrap value greater than 60 was labelled at each node in the tree.

#### Gene expression analysis

Total RNA was extracted using the HiPure HP Plant RNA Mini Kit (Magen, Guangzhou, China) according to the manufacturer’s protocol. RNA samples were reverse transcribed into cDNA using the Maxima H Minus First-Strand cDNA Synthesis Kit (Thermo Scientific, Shanghai, China). The qRT-PCR was performed with SYBR Green PCR Master Mix (Applied Biosystems, American), and amplification was performed on ABI 7900 Fast Real Time System (PE; Applied Biosystems). The elongation factor (*EF1α*) was used as the endogenous gene of citrus, while Ubiquitin (*UBQ*) was selected as the endogenous gene of tobacco. The used primers were listed in S4 Table. The relative gene expression was calculated using 2^−ΔΔCt^ method.

#### Transcriptome data analysis

Total RNA was extracted from sterile water- or *Xcc* (10^8^ CFU/ml)- inoculated Atalantia and sweet orang leaves at 6 h, 24 and 48 h after inoculation for RNA sequencing. The RNA-seq reads were mapped to the reference genome of sweet orange by HISAT2 [48]. The mapping results were transformed and sorted by samtools [49]. Gene expression values were normalized as Fragments Per Kilobase per Million (FPKM) by Cufflinks package [50]. The genes with P < 0.01 and an absolute value (fold change) log2 ratio ≥ 1 were defined as differentially expressed genes (DEGs). DEGs from the RNA seq data were analyzed and presented in S3 Table. Gene ontology term (GO) enrichment analysis was performed using Agrigo (https://www.Agrigo.com/) with FDR < 0.05. Our RNA-seq data were submitted to NCBI with genbank accession numbers of PRJNA612768 and PRJNA612769.

#### Molecular cloning and sequence analysis of *TFIIAγ* and *LOB1*

The *TFIIAγ* gene sequences were cloned from the cDNA of the ten citrus varieties (Murraya exotica, Atalantia, Citron, mangshan mandarin, Ponkan, Trifoliate orange, Eureka lemon, purple pummelo, guanxi pummelo, Sweet orange). Gene *AbLOB1* and *CsLOB1* and their promoter sequences were amplified from the cDNA and DNA of Atalantia and sweet orange. The primers used for amplification were listed in S4 Table. The gene ID of *AbTFIIAγ* was sb14580, and that of *CsTFIIAγ* gene was Cs3g16970 in the citrus genome database (http://citrus.hzau.edu.cn/cgi-bin/orange/blast). Sequence alignment of *TFIIAγ* and *LOB1* was conducted by ClustalW2 and GENEDOC software.

#### Homology modeling of TFIIAγ

The structures of AbTFIIAγ and CsTFIIAγ were homology modeled using SWISS-MODEL online service with default parameters (https://swissmodel.expasy.org/). The crystal structure of transcription initiation factor IIA from human HsTFIIA (PDB ID: 5M4S) was used as the template. AbTFIIAγ and CsTFIIAγ exhibited 53% and 49% sequence identity with the HsTFIIA template, respectively. The structures of AbTFIIAγ and CsTFIIAγ were generated by PyMOL.

#### Luciferase complementation assay

The full-length coding sequence (CDS) of *TFIIAγ* without stop codons was cloned into vector JW-like-771-nLuc, and sequence TFBi/TFBii were cloned into vector JW-like-772-cLuc. The primers were listed in S4 Table. All the vectors were transformed into *Agrobacterium tumefacien* strain GV3101. Then, the *Agrobacterium* strain carrying the two constructed reporter vectors (JW-like-771-nLuc and JW-like-772-cLuc at a ratio of 1:1) was infiltrated into tobacco leaves at OD 600 nm = 0.8. The images of luciferase signal were taken at 2 d via a charge coupled device camera (LB985 NightSHADE) from the Key Laboratory of Horticultural Plant Biology, Ministry of Education.

#### Pull-down assay

The full-length *TFIIAγ* and TFBs were fused into GST-tagged PGEX-6p and HIS-tagged PET-32a, respectively. The proteins with GST-tag or His-tag were expressed in *E*.*coli* BL21 strain in vitro and purified with a GST purification kit (Sangon Biotech, Shanghai, China) or Ni-NTA purification kit (Sangon Biotech, Shanghai, China). Both types of proteins were purified following the manufacturer’s protocol. The GST-tagged proteins were incubated with Mag-Beads GST fusion protein purification kit (Sangon Biotech, Shanghai, China) for two hours, and washed four times. After one-hour incubation of His-tagged protein, the Mag-Beads were eluted and target protein was separated. The eluted proteins were tested with anti-GST (Smart-lifesciences, Changzhou, China) or anti-His (Smart-lifesciences, Changzhou, China) by using western blotting.

#### *CsTFIIAγ* silencing assay

*CsTFIIAγ* gene sequence (174 bp from the ATG start codon) was cloned from sweet orange cDNA and fused into vector pk7GWIWG2D (provided by professor Chunying Kang of Key Laboratory of Horticultural Plant Biology). The self-complementary RNAi vector pk7GWIWG2D had the selection tag eGFP controlled by 35S promoter. The vector CsTFIIAγ-pk7GWIWG2D was transformed into *Agrobacterium tumefaciens* strain EHA105. The resultant *Agrobacterium* was further transformed into sweet orange epicotyl explants, as previously reported [51,52].

#### Vector construction

*NbTFIIAγ* (174 bp from ATG start codon) and *NbPDS* (369 bp of the coding sequence) were amplified from the *Nicotiana benthamiana* genome and fused into the tobacco pTRV2 vector. The complete CDS of *AbTFIIAγ* and *CsTFIIAγ* was amplified and fused into the overexpression vector PK7WG2D. Promoter *CsLOB1* and *AbLOB1* were amplified (296 bp upstream from *LOB1* coding sequence) and fused into uidA (β-glucuronidase (GUS) reporter gene vector pKGWFS7. The *pthA4* was amplified from *X*. *citri* strain 3213 genome and transferred into the PBI121 vector. Primer information was presented in S4 Table. All the constructs were transferred into GV3101 and suspended in the solution containing 10 mM MgCl_2_, 10 mM Mes (pH 5.6) and 100 μM acetosyringone.

#### Virus-induced gene silencing (VIGS) and *AbTFIIAγ* functional analysis

To investigate the putative function of *AbTFIIAγ*, VIGS assay was performed according to previous reports [53]. Phytoene desaturase (*PDS*) gene was used as the positive control. The mixed *Agrobacterium* GV3101 strains containing pTRV1and pTRV2 or pTRV1 and *NbTFIIAγ-pTRV* vectors were infiltrated into three-week-old tobacco, respectively. The inoculated tobacco was then transferred to a culture room and incubated at 22°C in the dark for two days, and then exposed to the cycle of 16-hour light and 8-hour darkness for one week. Subsequently, the plants were cultured at 25°C in the cycle of 16-hour light and 8-hour darkness for one week. When an obvious albino phenotype was observed, *Agrobacterium* GV3101 strains containing the 35S:*pthA4* and *pCsLOB1*:*GUS* mixed with 35S:*CsTFIIAγ* or 35S:*AbTFIIAγ*, or empty vector were co-infiltrated into *NbTFIIAγ-*silenced tobacco. Samples were collected 2 days after co-infiltration for gene expression and GUS activity analysis.

For a further analysis of *AbTFIIAγ* gene function, we complemented *AbTFIIAγ* into *CsTFIIAγ-*silenced citrus line (RNAi-1). In brief, the overexpression vector 35S:*AbTFIIAγ*, 35S:*CsTFIIAγ*, and empty vector PK7WG2D were separately transferred into *CsTFIIAγ*-silenced sweet orang leaves. Five hours later, *Xcc* was infiltrated at the same area at an OD 600nm = 0.3, as previously described [10]. Gene expression was analyzed 4 days after *Xcc* inoculation.

#### GUS activity assay

Firstly, GUS activity assay was performed in tobacco, vector 35S:*pthA4* and empty vector mixed with *pAbLOB1*:*GUS* or *pCsLOB1*:*GUS* were co-injected into tobacco leaves, respectively. The treated leaves were collected for GUS staining and GUS activity assay at day 2 after co-injection. We further analyzed the GUS activity in sweet orang leaves mediated by *Xcc*. Vector *pAbLOB1*:*GUS* or *pCsLOB1*:*GUS* was injected into sweet orange leaves, as previously reported [10]. Samples were collected at day 4 after vector injection. GUS staining assay was performed with GUS staining kit (MREDA, Shanghai, China) according to the manufacturer’s protocol with minor modifications. GUS activity assay was performed with the GUS activity assay kit (FCNCS, Nanjing, China) according to the manufacturer’s protocol.

## Supporting information

S1 Fig Bacterial growth and disease lesion area of ten citrus varieties. (A) Bacterial growth of 10 citrus varieties at 1 and 7 d after *Xcc* (10^8^ CFU/ml) inoculation. (B) Disease lesion area of 10 citrus varieties at 12 d after inoculation. Disease lesion area was calculated by ImageJ 2.0. Note: Wild mandarin: mangshan mandarin; Cultivated mandarin: Ponkan; Lemon: Eureka lemon; Wild pummelo: purple pummelo; Cultivated pummelo: guanxi pummelo. Error bars indicate standard deviation of three independent replicates.
(TIF)

S2 Fig qRT-PCR validation of selected DEGs in Atalantia and sweet orange after inoculation. (A) Three DEGs: pathogenesis-related gene 4 (*PR4*), quinone oxidoreductase, and repressor GA3 (*RGA3*). (B) Three DEGs: cytochrome P450 (*CYP76C4*), RED elongated 1 (*RED1*), and gibberellin 2-oxidase (*GA2OX1*). The treatment group was inoculated with *Xcc*, and the control was inoculated with sterile water at 6 h, 24 h, and 48 h. All the gene expressions were normalized according to the gene expression of the sweet orange control group at 6 h post inoculation. The relative expression level was calculated by 2^-△△Ct^ method with *EF1a* as the reference gene. Error bars indicate standard deviation of three independent repetitions. (C) Correlations between qRT-PCR gene expression and RNA-seq data. A linear regression line (green). Correlation coefficients and y = x line (black, dotted) are also shown in each panel. The x and y axes represent qRT-PCR Log2 (fold change) and RNA-seq Log2 (fold change), respectively. For RNA-seq data, fold-changes of gene expression level (fragments per kilobase of transcript per million mapped reads, FPKMs) were normalized to the FPKM of the control (inoculation with sterile water at 6 h, 24 h, and 48 h). For qRT-PCR data gene expression levels were calculated by 2^-△△Ct^ method with *EF1a* as the reference gene.
(TIF)

S3 Fig Amino acid sequence alignment of TFIIAγ from the ten citrus varieties. These 10 varieties include Wild mandarin: mangshan mandarin; Cultivated mandarin: Ponkan; Lemon: Eureka lemon; Wild pummelo: purple pummelo; Cultivated pummelo: guanxi pummelo, and other varieties. The red box represents the 81th, 87th, and 90th amino acid, respectively. The sequence alignment was conducted by ClustalW2 and GENEDOC software.
(TIF)

S4 Fig Homology modeling of AbTFIIAγ and CsTFIIAγ proteins. (A) Homology modeling of AbTFIIAγ protein. (B) Homology modeling of mutated AbTFIIAγ^V81L^ proteins. (C) Comparison of AbTFIIAγ and mutated AbTFIIAγ^V81L^ proteins. (D) Homology modeling of CsTFIIAγ protein. (E) Homology modeling of mutated CsTFIIAγ^L81V^ proteins. (F) Comparison of CsTFIIAγ and mutated CsTFIIAγ^L81V^ proteins. The structures of TFIIAγ proteins were homology modeled using SWISS-MODEL online service with default parameters.
(TIF)

S5 Fig Gene expression level of *NbTFIIAγ*, *AbTFIIAγ*, and *CsTFIIAγ* in *NbTFIIAγ-*silenced line of tobacco. Gene expression was detected after 2 days of transient expression. Data from three independent replicates were expressed as mean ± SD. Different letters above the bars represent significant differences (P < 0.05) in Duncan’s multiple range test.
(TIF)

S6 Fig Alignment of *AbLOB1* and *CsLOB1* promoter and amino acid sequences. (A) Sequence alignment of *AbLOB1* and *CsLOB1* promoter. The promoter *pAbLOB1* and *pCsLOB1* represent *AbLOB1* and *CsLOB1* promoter (296 bp upstream from *LOB1* coding sequence), respectively. The red box represents effector binding element (EBE). (B) Alignment of the predicted amino acid sequence of AbLOB1 and CsLOB1. Sequence alignment was performed using ClustalW2 and GENEDOC software.
(TIF)

S7 Fig Relative expression level of *LOB1* with different treatments. Atalantia and Sweet orange leaves were respectively treated with *Xcc* (10^8^ CFU/mL), Xcc306*ΔpthA4* (10^8^ CFU/mL), sterile water, and a control group was subjected to no treatment. RNA was extracted 48 h after inoculation. Relative expression level was calculated by the method of 2^-△△Ct^ with *EF1a* as the reference gene. Error bars indicate standard deviation of three independent tests.
(TIF)

S1 Table Number of paired-end reads counted by Illumina sequencing technology. Note: ATL and SWO represent Atalantia and sweet orange, respectively. The treatment group and control group were inoculated with *Xcc* and sterile water at 6 h, 24 h, and 48 h.
(DOCX)

S2 Table List of 8 overlapping differentially expressed genes 48 h after *Xcc* inoculation in Atalantia and sweet orange. Note: fold-changes of gene expression level (fragments per kilobase of transcript per million mapped reads, FPKMs) were normalized to the FPKM of the control (inoculation with sterile water at 48 h). ‘-’ indicates down-regulation.
(DOCX)

S3 Table Differentially expressed genes (DEGs) in Atalantia and sweet orange after *Xcc* inoculation. ATL and SWO represent Atalantia and sweet orange, respectively. T6, T24, and T48 represent the treatment time points (inoculation with *Xcc* at 6 h, 24 h and 48 h); C6, C24, C48 represent the control (inoculation with sterile water at 6 h, 24 h and 48 h).
(XLSX)

S4 Table Primer sequences used in this study. (DOCX)
