# Supplementary figures and images for: Natural variations of TFIIAγ gene and LOB1 promoter contribute to citrus canker disease resistance in Atalantia buxifolia
Source: PLoS Genet. 2021 Jan 25;17(1):e1009316. doi: 10.1371/journal.pgen.1009316 (PMC7861543; doi:10.1371/journal.pgen.1009316)

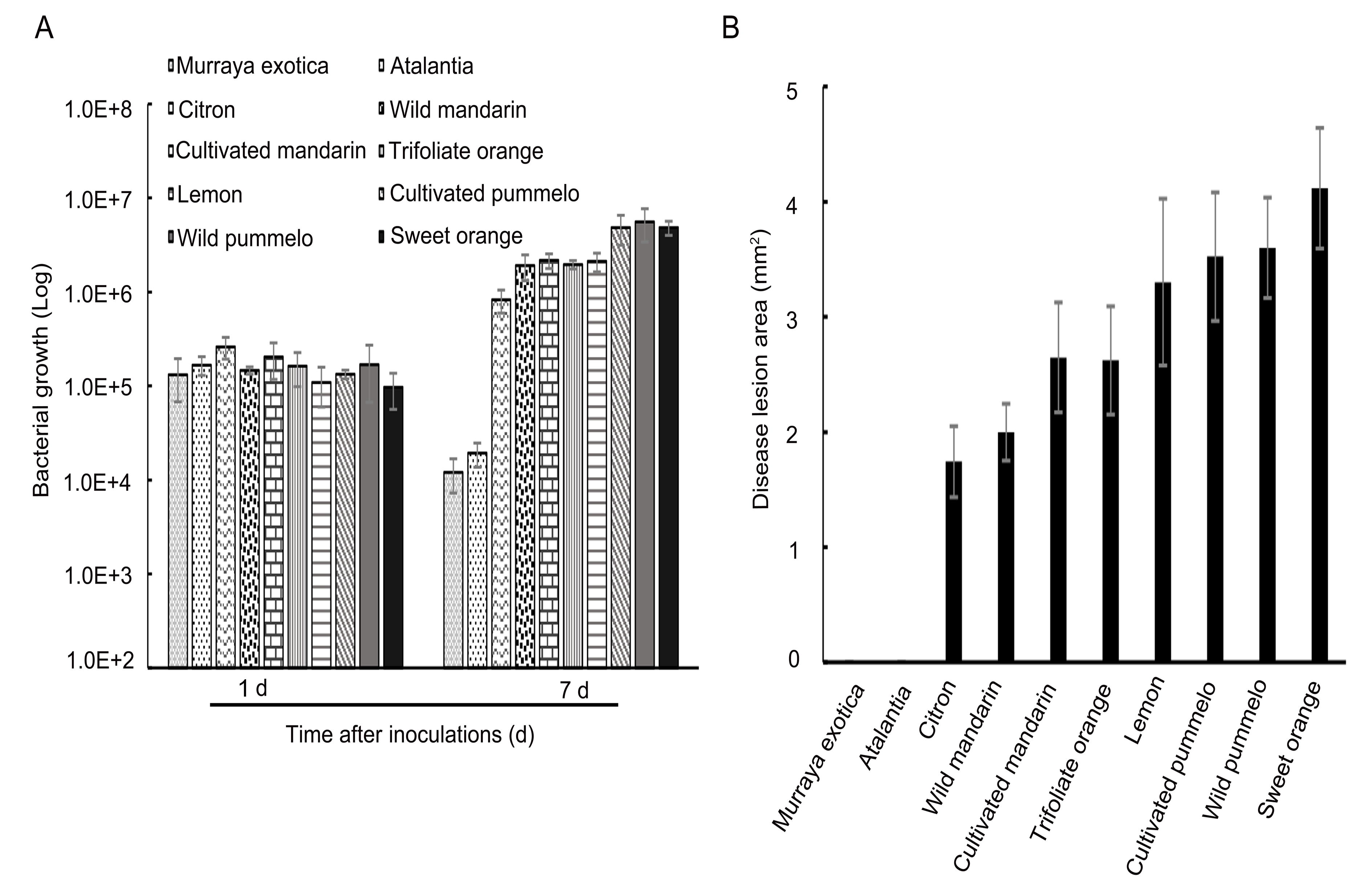

Supplement: S1 Fig — (A) Bacterial growth of 10 citrus varieties at 1 and 7 d after Xcc (108 CFU/ml) inoculation. (B) Disease lesion area of 10 citrus varieties at 12 d after inoculation. Disease lesion area was calculated by ImageJ 2.0. Note: Wild mandarin: mangshan mandarin; Cultivated mandarin: Ponkan; Lemon: Eureka lemon; Wild pummelo: purple pummelo; Cultivated pummelo: guanxi pummelo. Error bars indicate standard deviation of three independent replicates. (TIF) [file pgen.1009316.s001.tif]

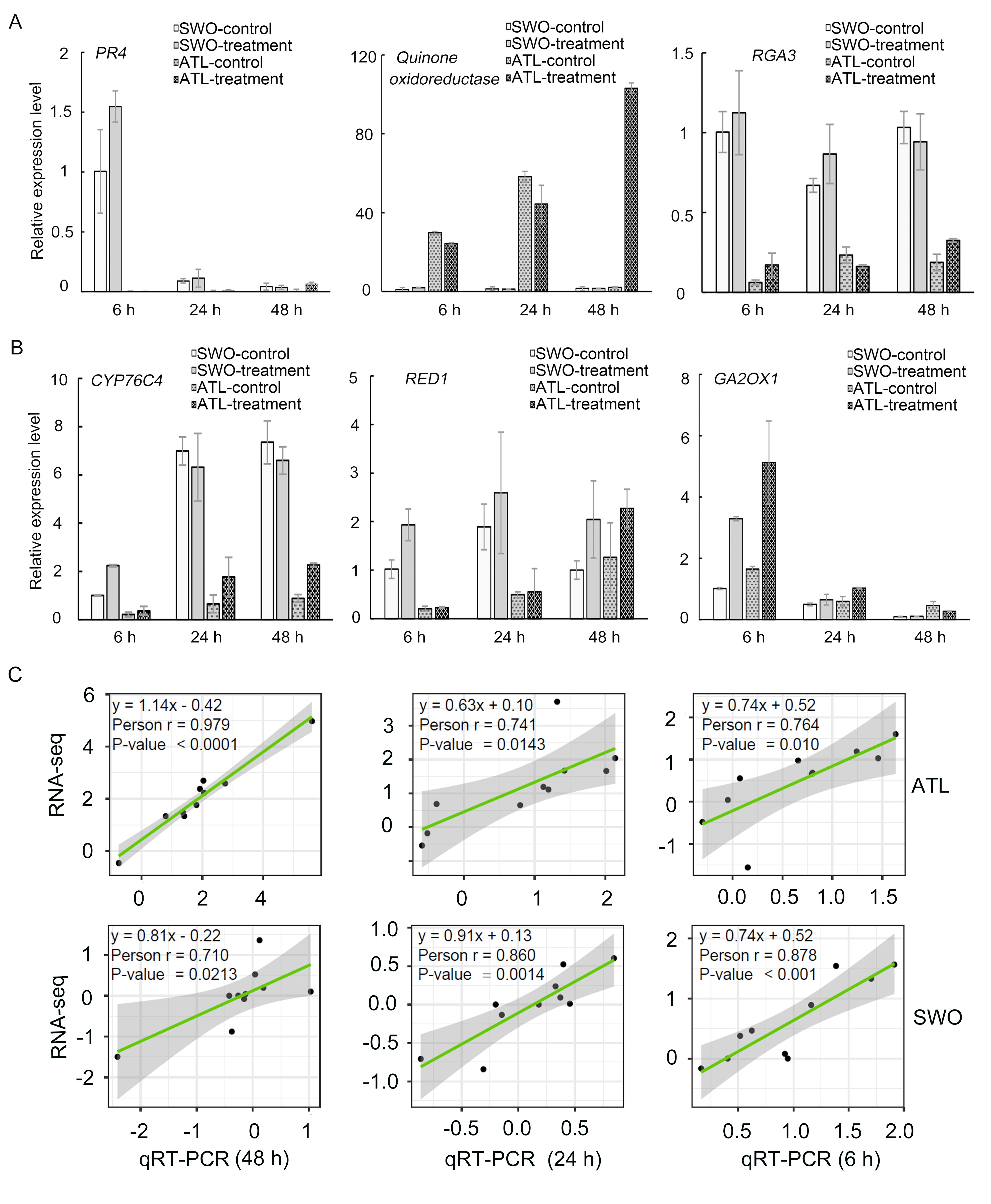

Supplement: S2 Fig — (A) Three DEGs: pathogenesis-related gene 4 (PR4), quinone oxidoreductase, and repressor GA3 (RGA3). (B) Three DEGs: cytochrome P450 (CYP76C4), RED elongated 1 (RED1), and gibberellin 2-oxidase (GA2OX1). The treatment group was inoculated with Xcc, and the control was inoculated with sterile water at 6 h, 24 h, and 48 h. All the gene expressions were normalized according to the gene expression of the sweet orange control group at 6 h post inoculation. The relative expression level was calculated by 2-△△Ct method with EF1a as the reference gene. Error bars indicate standard deviation of three independent repetitions. (C) Correlations between qRT-PCR gene expression and RNA-seq data. A linear regression line (green). Correlation coefficients and y = x line (black, dotted) are also shown in each panel. The x and y axes represent qRT-PCR Log2 (fold change) and RNA-seq Log2 (fold change), respectively. For RNA-seq data, fold-changes of gene expression level (fragments per kilobase of transcript per million mapped reads, FPKMs) were normalized to the FPKM of the control (inoculation with sterile water at 6 h, 24 h, and 48 h). For qRT-PCR data gene expression levels were calculated by 2-△△Ct method with EF1a as the reference gene. (TIF) [file pgen.1009316.s002.tif]

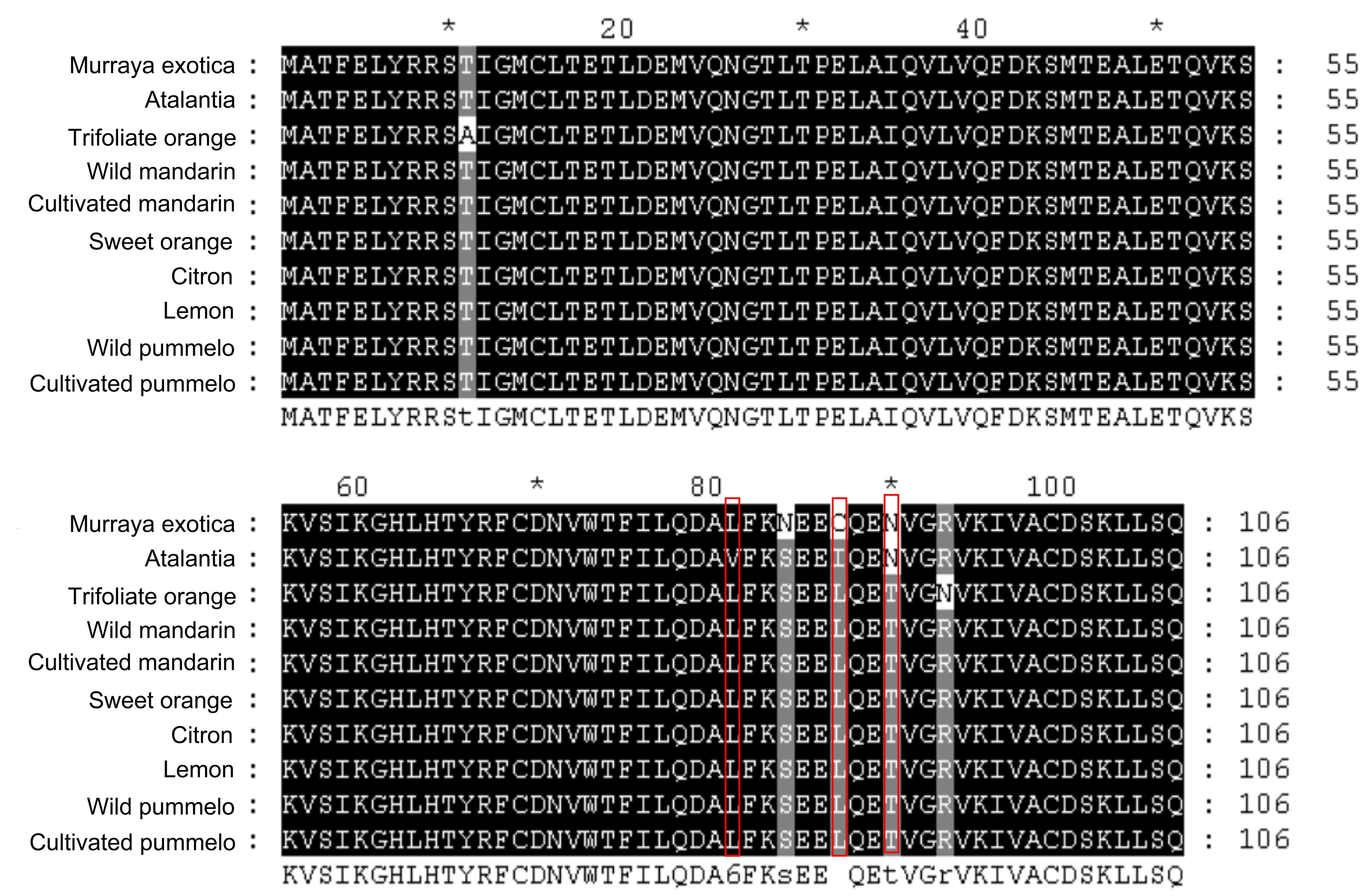

Supplement: S3 Fig — These 10 varieties include Wild mandarin: mangshan mandarin; Cultivated mandarin: Ponkan; Lemon: Eureka lemon; Wild pummelo: purple pummelo; Cultivated pummelo: guanxi pummelo, and other varieties. The red box represents the 81th, 87th, and 90th amino acid, respectively. The sequence alignment was conducted by ClustalW2 and GENEDOC software. (TIF) [file pgen.1009316.s003.tif]

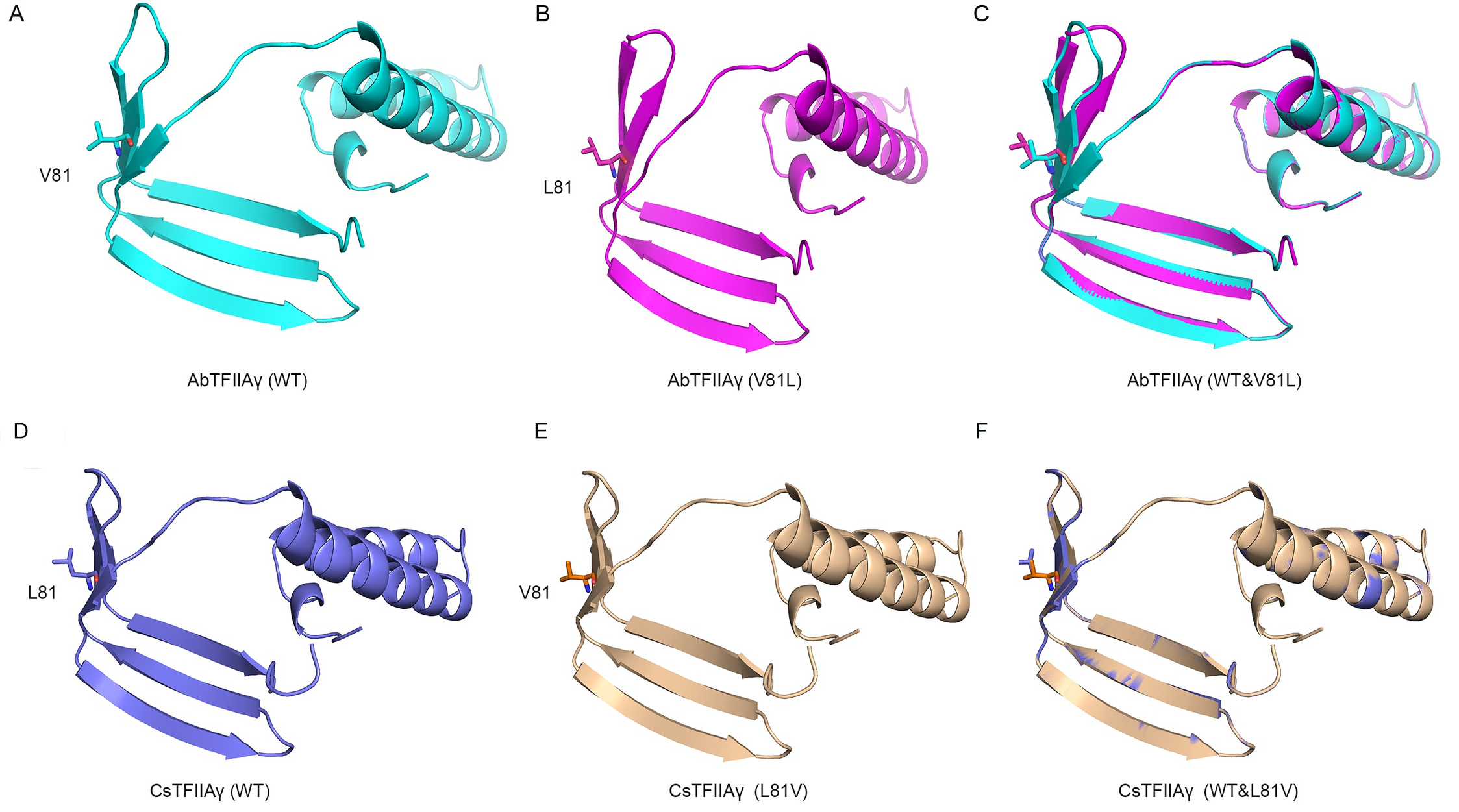

Supplement: S4 Fig — (A) Homology modeling of AbTFIIAγ protein. (B) Homology modeling of mutated AbTFIIAγV81L proteins. (C) Comparison of AbTFIIAγ and mutated AbTFIIAγV81L proteins. (D) Homology modeling of CsTFIIAγ protein. (E) Homology modeling of mutated CsTFIIAγL81V proteins. (F) Comparison of CsTFIIAγ and mutated CsTFIIAγL81V proteins. The structures of TFIIAγ proteins were homology modeled using SWISS-MODEL online service with default parameters. (TIF) [file pgen.1009316.s004.tif]

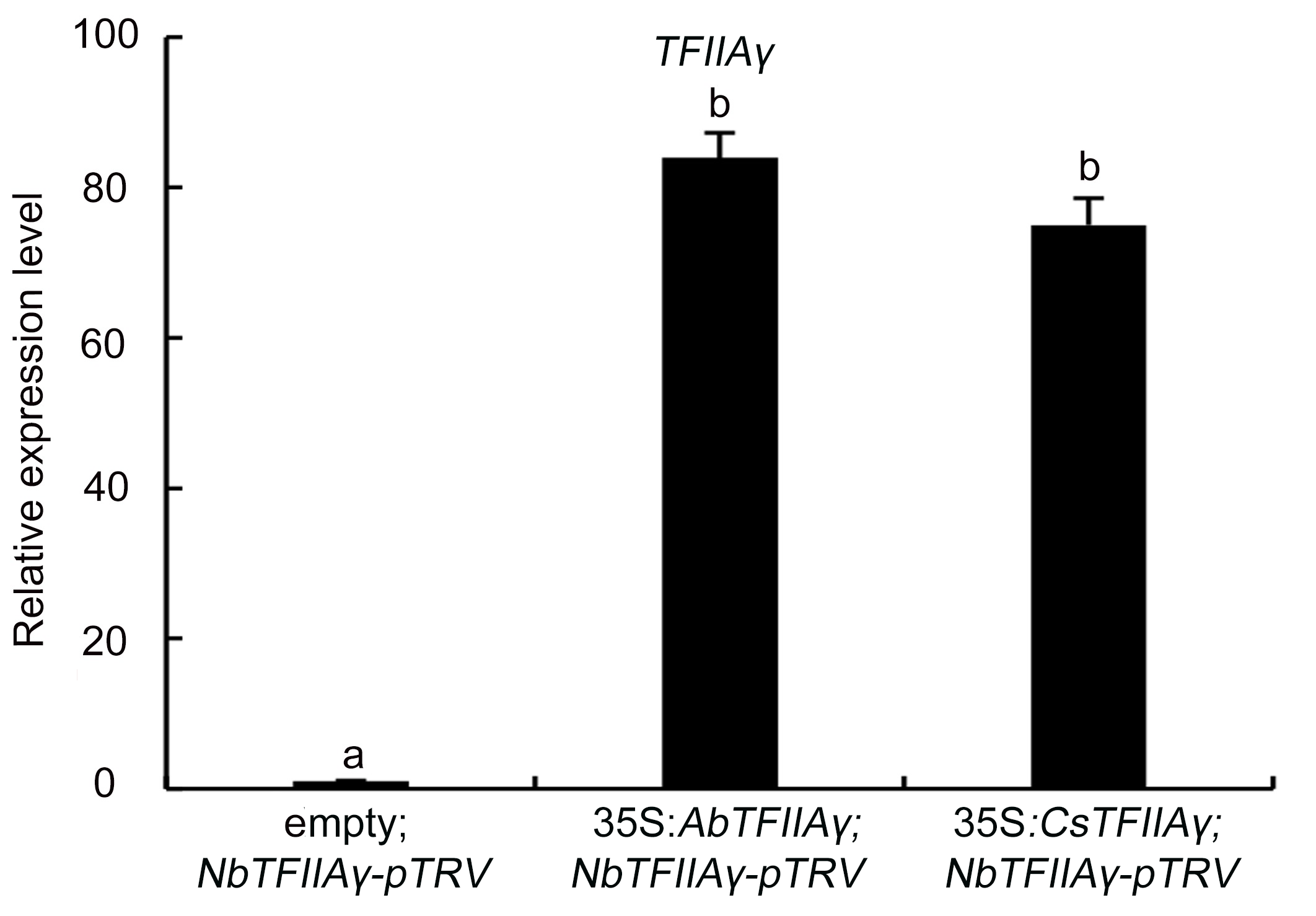

Supplement: S5 Fig — Gene expression was detected after 2 days of transient expression. Data from three independent replicates were expressed as mean ± SD. Different letters above the bars represent significant differences (P < 0.05) in Duncan’s multiple range test. (TIF) [file pgen.1009316.s005.tif]

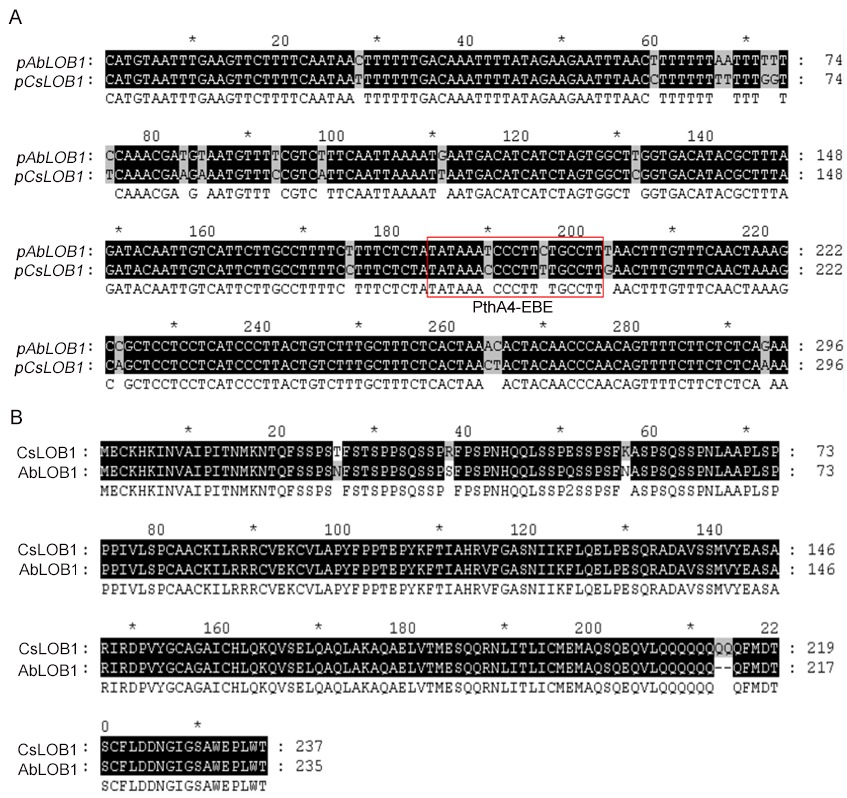

Supplement: S6 Fig — (A) Sequence alignment of AbLOB1 and CsLOB1 promoter. The promoter pAbLOB1 and pCsLOB1 represent AbLOB1 and CsLOB1 promoter (296 bp upstream from LOB1 coding sequence), respectively. The red box represents effector binding element (EBE). (B) Alignment of the predicted amino acid sequence of AbLOB1 and CsLOB1. Sequence alignment was performed using ClustalW2 and GENEDOC software. (TIF) [file pgen.1009316.s006.tif]

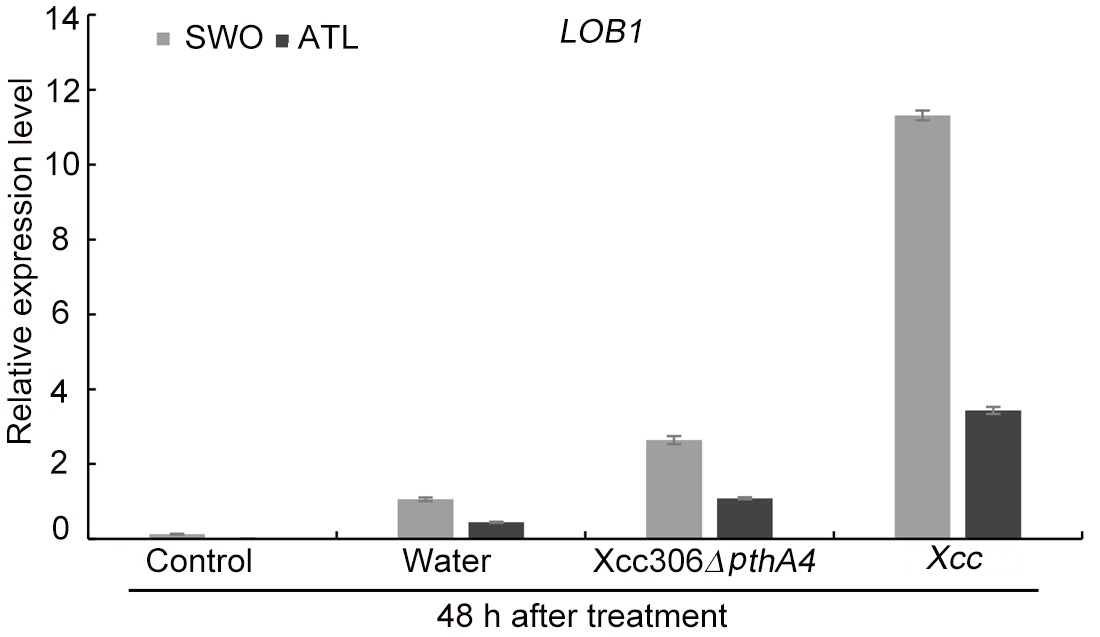

Supplement: S7 Fig — Atalantia and Sweet orange leaves were respectively treated with Xcc (108 CFU/mL), Xcc306ΔpthA4 (108 CFU/mL), sterile water, and a control group was subjected to no treatment. RNA was extracted 48 h after inoculation. Relative expression level was calculated by the method of 2-△△Ct with EF1a as the reference gene. Error bars indicate standard deviation of three independent tests. (TIF) [file pgen.1009316.s007.tif]
