# Supplementary material for: Natural variations of TFIIAγ gene and LOB1 promoter contribute to citrus canker disease resistance in Atalantia buxifolia
Source: PLoS Genet. 2021 Jan 25;17(1):e1009316. doi: 10.1371/journal.pgen.1009316 (PMC7861543; doi:10.1371/journal.pgen.1009316)
Supplement: S1 Table — Note: ATL and SWO represent Atalantia and sweet orange, respectively. The treatment group and control group were inoculated with Xcc and sterile water at 6 h, 24 h, and 48 h. (DOCX) [file pgen.1009316.s008.docx]

**S1 Table. Numbers of paired-end reads counted by Illumina sequencing technology.**

| **Library** | **Number of read pairs** | **Read length** | **Total base** | |
| --- | --- | --- | --- | --- |
| SWO-control6-1 | 30,203,147 | 150 | | 9,060,944,100 |
| SWO-control6-2 | 26,554,915 | 150 | | 7,966,474,500 |
| SWO-control24-1 | 39,776,075 | 150 | | 11,932,822,500 |
| SWO-control24-2 | 34,796,673 | 150 | | 10,439,001,900 |
| SWO-control48-1 | 30,173,430 | 150 | | 9,052,029,000 |
| SWO-control48-2 | 32,323,789 | 150 | | 9,697,136,700 |
| SWO-treatment6-1 | 28,621,220 | 150 | | 8,586,366,000 |
| SWO-treatment6-2 | 38,849,952 | 150 | | 11,654,985,600 |
| SWO-treatment24-1 | 25,447,852 | 150 | | 7,634,355,600 |
| SWO-treatment24-2 | 31,040,829 | 150 | | 9,312,248,700 |
| SWO-treatment48-1 | 31,226,942 | 150 | | 9,368,082,600 |
| SWO-treatment48-2 | 41,732,345 | 150 | | 12,519,703,500 |
| ATL-control6-1 | 28,019,318 | 150 | | 8,405,795,400 |
| ATL-control6-2 | 33,913,256 | 150 | | 10,173,976,800 |
| ATL-control24-1 | 27,738,006 | 150 | | 8,321,401,800 |
| ATL-control24-2 | 32,068,185 | 150 | | 9,620,455,500 |
| ATL-control48-1 | 31,610,960 | 150 | | 9,483,288,000 |
| ATL-control48-2 | 30,657,796 | 150 | | 9,197,338,800 |
| ATL-treatment6-1 | 30,988,249 | 150 | | 9,296,474,700 |
| ATL-treatment6-2 | 36,122,427 | 150 | | 10,836,728,100 |
| ATL-treatment24-1 | 25,966,904 | 150 | | 7,790,071,200 |
| ATL-treatment24-2 | 40,794,033 | 150 | | 12,238,209,900 |
| ATL-treatment48-1 | 28,367,751 | 150 | | 8,510,325,300 |
| ATL-treatment48-2 | 35,045,333 | 150 | | 10,513,599,900 |
| total | 772,039,387 |  | | 231,611,816,10 |

Note: ATL and SWO represent Atalantia and sweet orange, respectively. The treatment group and control group were inoculated with *Xcc* and sterile water at 6 h, 24 h, and 48 h.
