# Supplementary material for: Natural variations of TFIIAγ gene and LOB1 promoter contribute to citrus canker disease resistance in Atalantia buxifolia
Source: PLoS Genet. 2021 Jan 25;17(1):e1009316. doi: 10.1371/journal.pgen.1009316 (PMC7861543; doi:10.1371/journal.pgen.1009316)
Supplement: S2 Table — Note: fold-changes of gene expression level (fragments per kilobase of transcript per million mapped reads, FPKMs) were normalized to the FPKM of the control (inoculation with sterile water at 48 h). ‘-’ indicates down-regulation. (DOCX) [file pgen.1009316.s009.docx]

**S2 Table. List of the 8 overlapping differentially expressed genes 48 h after *Xcc* inoculation in Atalantia and sweet orange.**

| **Term**  **Target description** | **Atalantia Sweet orange**  **Gene ID Gene ID** | | **Fold change 48 h inoculation**  **Atalantia Sweet orang** | |
| --- | --- | --- | --- | --- |
| Multidrug and toxin extrusion protein 1  LOB domain-containing protein 1  18 kDa seed maturation protein  Adenylate isopentenyltransferase 5, chloroplastic  Abscisic acid 8'-hydroxylase 4  Homogentisate phytyltransferase 1, chloroplastic  Probable pectate lyase 5  Endoglucanase 9 | sb14670  sb24031  sb37413  sb19528  sb36614  sb16820  sb32837  sb16426 | Cs3g_pb024130  Cs7g_pb020690  Cs6g_pb014410  Cs9g_pb006030  Cs8g_pb002760  Cs4g_pb022160  Cs2g_pb020970  Cs9g_pb006510 | 2.7  4.6  27.4  11.0  -2.6  2.3  -2.4  -2.7 | 10.4  8.7  2.6  2.8  -4.1  -2.7  2.6  2.8 |

Note: fold-changes of gene expression level (fragments per kilobase of transcript per million mapped reads, FPKMs) were normalized to the FPKM of the control (inoculation with sterile water at 48 h). ‘-’ indicates down-regulation.
