# Supplementary material for: Natural variations of TFIIAγ gene and LOB1 promoter contribute to citrus canker disease resistance in Atalantia buxifolia
Source: PLoS Genet. 2021 Jan 25;17(1):e1009316. doi: 10.1371/journal.pgen.1009316 (PMC7861543; doi:10.1371/journal.pgen.1009316)
Supplement: S4 Table — (DOCX) [file pgen.1009316.s011.docx]

**S4 Table. Primer sequences used in this study.**

| Primer ID | Sequence of primers (5’-3’) | Purpose |
| --- | --- | --- |
| TFIIAγ-F | AGCCCCAAAATCTCTCCGCCCAA | cDNA fragment amplifying of ten citrus varieties |
| TFIIAγ-R | CCAGTTGAAAACAGTTGGCGGCT |  |
| TFIIAγ-pK7WG2D-F | AAAAAGCAGGCTTCATGGCGACGTTTGAGCTGTAT | Overexpression vector construction |
| TFIIAγ-pK7WG2D-R | AGAAAGCTGGGTGTCATTGTGATAGCAGCTTTGA |  |
| attB1-F | GGGACAAGTTTGTACAAAAAAGCAGGCT | Gateway vectors  construction |
| attB2-R | GGGGACCACTTTGTACAAGAAAGCTGGGT |  |
| TFIIAγ-pK7GWIWG2D-F | AAAAAGCAGGCTTCATGGCGACGTTTGAGC | Silencing vector construction |
| TFIIAγ-pK7GWIWG2D-R | AGAAAGCTGGGTGCTTCACCTGAGTTTC |  |
| TFIIAγ-JW771-F | ACGGGGGACGAGCTCGGTACCATGGCGACGTTTGAGCTG | Dual-luc vectors construction |
| TFIIAγ-JW771-R | GCGTACGAGATCTGGTCGACTTGTGATAGCAGCTTTGAG |  |
| TFBs-JW772-F | TACGCGTCCCGGGGCGGTACCATGAGCATTGTTGCCCAGTTATC |  |
| TFBs-JW772-R | ACGAAAGCTCTGCAGGTCGACCATCCCTGATGCCTGGAGGATAC |  |
| NbPDS-TRV2-F | GGTTACCGAATTCTCTAGACTGACGAGCTTTCGATGCAGTG | VIGS vectors construction |
| NbPDS-TRV2-R | GACGCGTGAGCTCGGTACCATATATGGACATTTATCACAGG |  |
| NbTFIIAγ-TRV2-F | GGTTACCGAATTCTCTAGAATGGCGACATTCGAGCTGTAC |  |
| NbTFIIAγ-TRV2-R | GACGCGTGAGCTCGGTACC GTCTTTACTTGACTCTCCAAAGC |  |
| pCsLOB-PkGWFS7-F | AAAAAGCAGGCTTCCATGTAATTTG | Promoter vectors construction |
| pCsLOB-PkGWFS7-R | AGAAAGCTGGGTGTTTTGAGAGAA |  |
| pAbLOB-pKGWFS7-F | AAAAAGCAGGCTTCCATGTAATTTGAAGT |  |
| pAbLOB-pKGWFS7-R | AGAAAGCTGGGTGTTCTGAGAGAA |  |
| PthA4-PBI121-F | GCTCTAGAATGGATCCCATTCGTTC | Overexpression vector construction |
| PthA4-PBI121-R | CGAGCTCTCACTGAGGCAATAG |  |
| TFIIAγ-PGEx6p-F | CCCCTGGGATCCCCGGAATTCATGGCGACGTTTGAGCTGT | Prokaryotic expression vectors construction |
| TFIIAγ-PGEx6p-R | GATGCGGCCGCTCGAGTCGACTTATTGTGATAGCAGCTTTGAG |  |
| TFBs-PET32a-F | GCTGATATCGGATCCGAATTCATGAGCATTGTTGCCCAGTTAT |  |
| TFBs-PET32a-R | TGCGGCCGCAAGCTTGTCGACCATCCCTGATGCCTGGAG |  |
| EF1a-F | GTAACCAAGTCTGCTGCCAAG | q-PCR |
| EF1a-R | GACCCAAACACCCAACACATT |  |
| q-TFIIAγ-F | TGAAGAGCAAGGTCTCCATTAAGG |  |
| q-TFIIAγ-R | TTCCTCACTCTTGAACAAAGCATCT |  |
| q-LOB1-F | TCCACCAACCGAACCATACA |  |
| q-LOB1-R | GGCACTTGCTTCATAGACCAT |  |
| q-TFB-F | GCATTTGATGACGCCATGAC |  |
| q-TFB-R | TCCCTGATGCCTGGAGGATA |  |
| q-NbTFIIAγ-F | GCCCAGAACTCGCCATTCAA |  |
| q-NbTFIIAγ-R | CAATCTTAACGCAGCTAACAG |  |
| UBQ-F | GGTGTTTCCAGTGGCGGACG |  |
| UBQ-R | TCCTCCCCTCAGCTACGGGGTAT |  |
| PR1-F | CTCACCCCAAGACTATGTCAATG |  |
| PR1-R | AAGGTTCTCGCCATACGGTCC |  |
| WIN2-F | GCAACTTATCACTTTTACAATCCTG |  |
| WIN2-R | TGTGGCTCTGTTAGTCACCCTTA |  |
| RPS5-F | ACAAGTGCGTCCAAGGTAGTTTT |  |
| RPS5-R | CCTCAAATAATCTCCAGGCATCT |  |
| Dirigent protein22-F | GATGAGCGAAGTCAGAGAAATGC |  |
| Dirigent protein22-R | TATCCTATCACAGCGTCCATTTG | qPCR |
| PR4-F | GGAGGTTCGGTCTTTGTGTGATT |  |
| PR4-R | TTGTCCAATGGGTCCGCCCGAAT |  |
| quinone oxidoreductase-F | TGGAAAATCAAGAAGGGAGTAGC |  |
| quinone oxidoreductase-R | ACACCAGCAGATTGAGTAAGC |  |
| RGA3-F | TCAAAGAAGAGGTTGGTTCGGTG |  |
| RGA3-R | AGTCTCCAGTATGTCTTCGGCGT |  |
| CYP76C4-F | GGCGAGGTGACCACAGTAGTA |  |
| CYP76C4-R | CCAGGTTGTTGAGACAGGCAG |  |
| RED1-F | CTTGGTGAAAGGATTGGACTA |  |
| RED1-R | CATCTCTGCTTTTGTTCTTTTTGGA |  |
| GA2OX1-F | GAAGCCATCAGGTTTTTCTCA |  |
| GA2OX1-R | AAGATACTCAACCCAACCCAC |  |
